# Supplementary material for: GelGenie: an AI-powered framework for gel electrophoresis image analysis
Source: Nat Commun. 2025 May 5;16:4087. doi: 10.1038/s41467-025-59189-0 (PMC12053679; doi:10.1038/s41467-025-59189-0)
Supplement: Supplementary file 1 — Supplementary Information [file 41467_2025_59189_MOESM1_ESM.pdf]

Supplementary Figures and Tables for:

"GelGenie: an AI-powered framework for gel electrophoresis image analysis"

Matthew Aquilina[1,2,3,4*], Nathan J. W. Wu[1], Kiros Kwan[1], Filip Bušić[1], James Dodd[1], Laura Nicolás-Sáenz[5], Alan O'Callaghan[5], Peter Bankhead[5,6,7], Katherine E. Dunn[1*]

[1]Institute for Bioengineering, School of Engineering, University of Edinburgh, Colin Maclaurin Road, The King's Buildings, Edinburgh, EH9 3DW, Scotland, UK.
[2]Deanery of Molecular, Genetic and Population Health Sciences, University of Edinburgh, Edinburgh, EH8 9AG, Scotland, UK.
[3]Current Address: Department of Cancer Biology, Dana-Farber Cancer Institute, Boston, MA 02215, Massachusetts, USA.
[4]Current Address: Wyss Institute for Biological Engineering, Harvard University, Boston, MA 02215, Massachusetts, USA.
[5]Centre for Genomic & Experimental Medicine, Institute of Genetics and Cancer, University of Edinburgh, Edinburgh, EH4 2XU, Scotland, UK.
[6]Edinburgh Pathology, Institute of Genetics and Cancer, University of Edinburgh, Edinburgh, EH4 2XU, Scotland, UK.
[7]CRUK Scotland Centre, Institute of Genetics and Cancer, University of Edinburgh, Edinburgh, EH4 2XU, Scotland, UK.

*Corresponding author(s). E-mail(s): matthew_aquilina@dfci.harvard.edu; k.dunn@ed.ac.uk;

A) **2_Thermo (low error gel)**

Original Image | Segmentation Map | GelAnalyzer Lane Plot

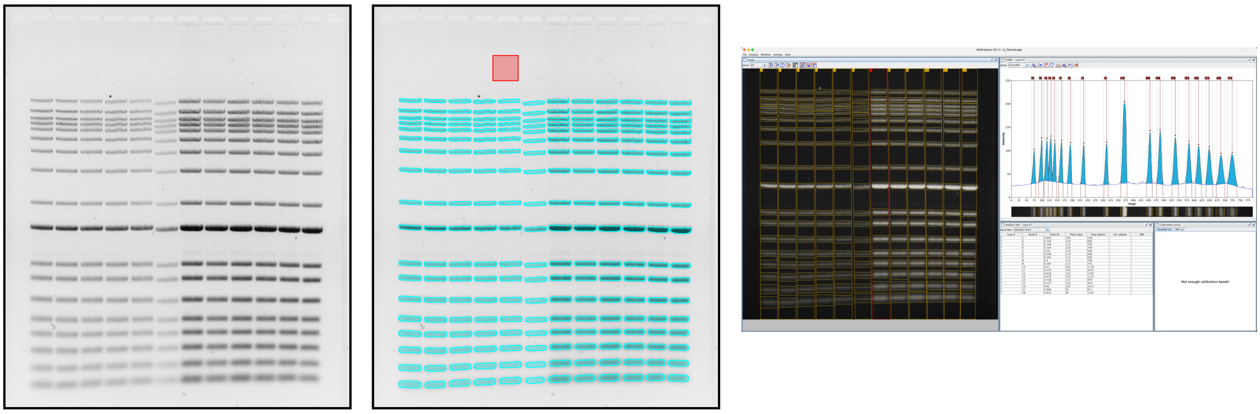

B) **10_Thermo (high error gel)**

Original Image | Segmentation Map | GelAnalyzer Lane Plot

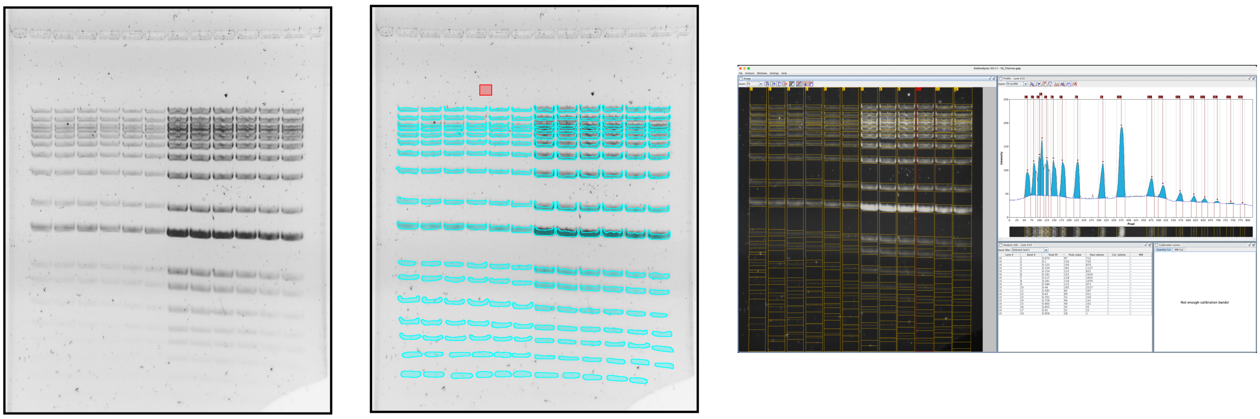

Supp. Fig. 1: Examples highlighting the diversity of the images generated for the ladder quantitation dataset analysed in Fig. 1. The red square in the segmentation maps indicates the region from which the global background was calculated. **A)** An example of a ladder image with low background and highly distinct/sharp bands. Accordingly, lane quantitation error for this image was low (≈10%) across almost all methods used. **B)** An example of a ladder image with very faint, low sharpness and highly overlapping bands. Accordingly, lane quantitation error for this image was high (≈35%+) across all methods used.

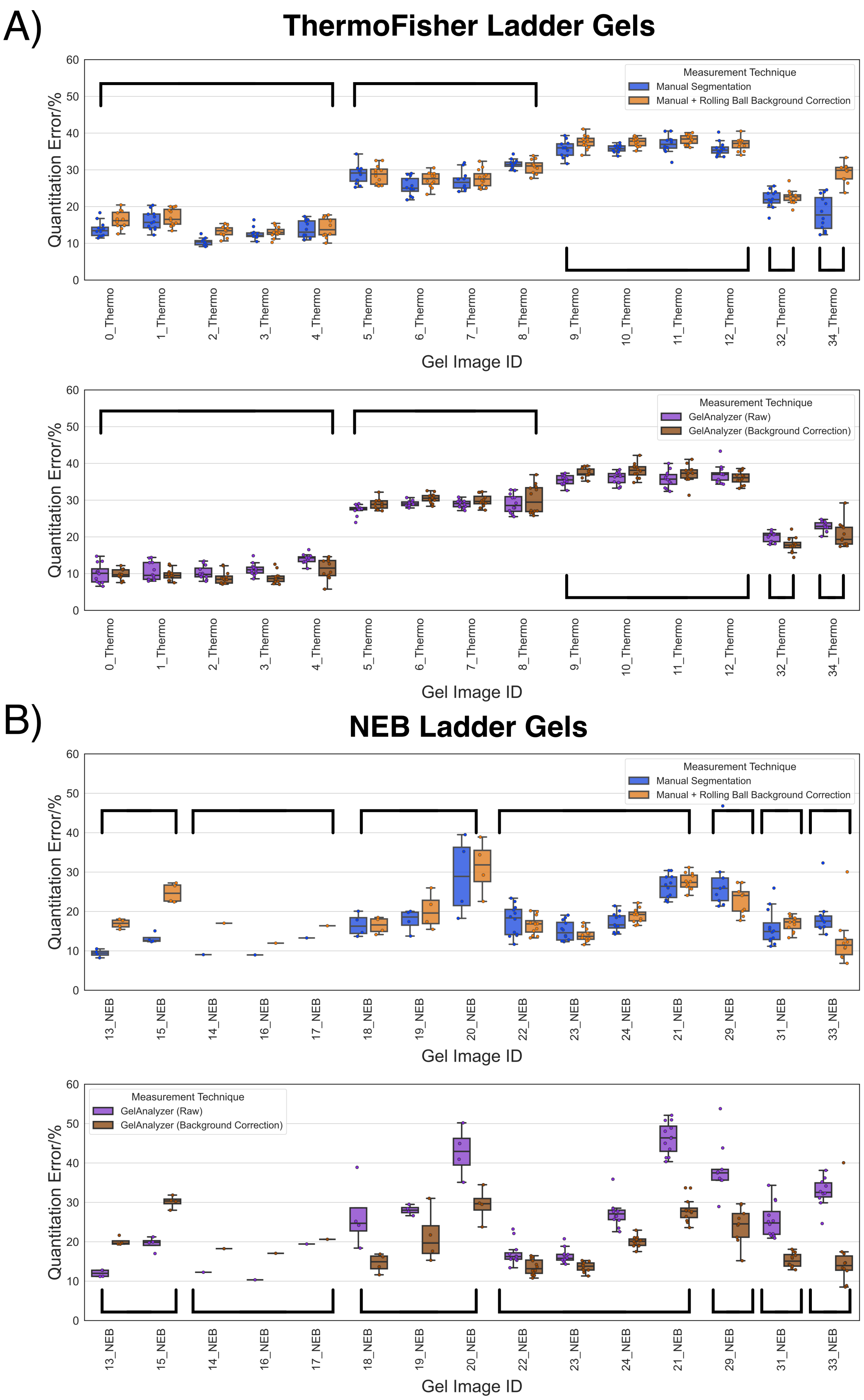

Supp. Fig. 2: The data from Figure 1B was rearranged into boxplots split by both ladder (**A)** for ThermoFisher and **B)** for NEB) and individual gel source image. The braces indicate which images came from the same source gel; each image in a group was taken with a different exposure time, with the time increasing from left to right. The data clearly showed that experimental conditions had the largest impact on quantification accuracy, with background correction having significantly less effect for the segmentation approach and a more pronounced effect for the GelAnalyzer results on the NEB ladder dataset. All plots display the median as a horizontal line within each box. The bounds of each box are defined by the lower quartile (25th percentile) and upper quartile (75th percentile). The whiskers extend to the most extreme data points within 1.5 times the interquartile range (IQR) from the quartiles. All individual points for each plot have been overlaid over each sample column.

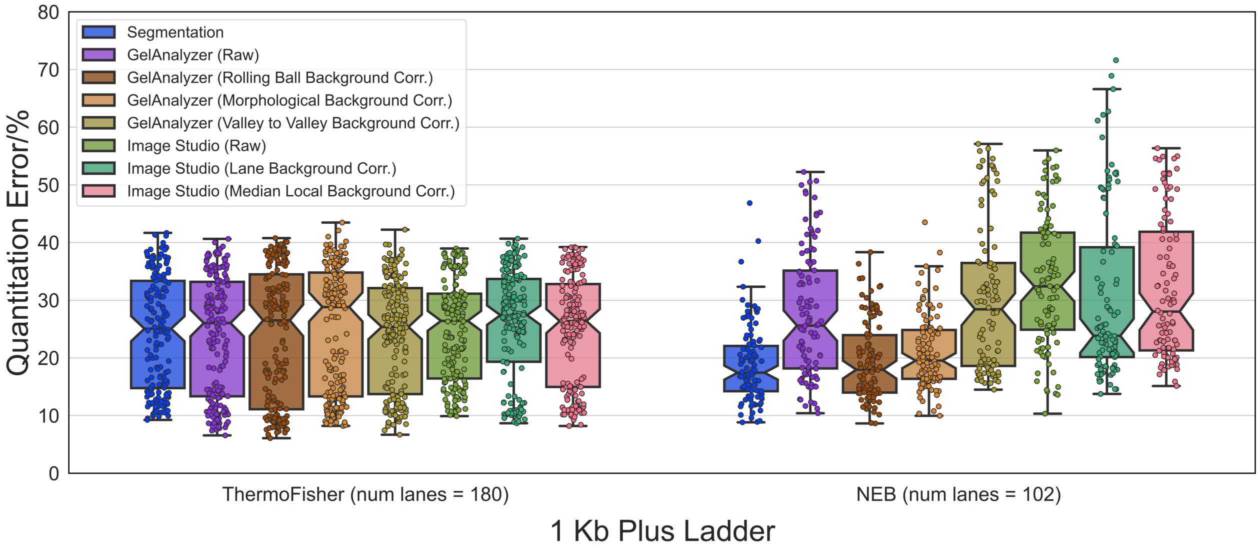

Supp. Fig. 3: Boxplots showing extended analyses on the dataset from Fig. 1 conducted using LI-COR's Image Studio and additional background correction methods available in GelAnalyzer. None of the additional methods appear to provide any significant benefit over segmentation, and in fact appear to perform worse on the NEB dataset. The box plots display the median as a horizontal line within each box. The bounds of each box are defined by the lower quartile (25th percentile) and upper quartile (75th percentile). The notches provide the 95% confidence interval around the median. The whiskers extend to the most extreme data points within 1.5 times the interquartile range (IQR) from the quartiles. All individual points for each plot have been overlaid over each box.

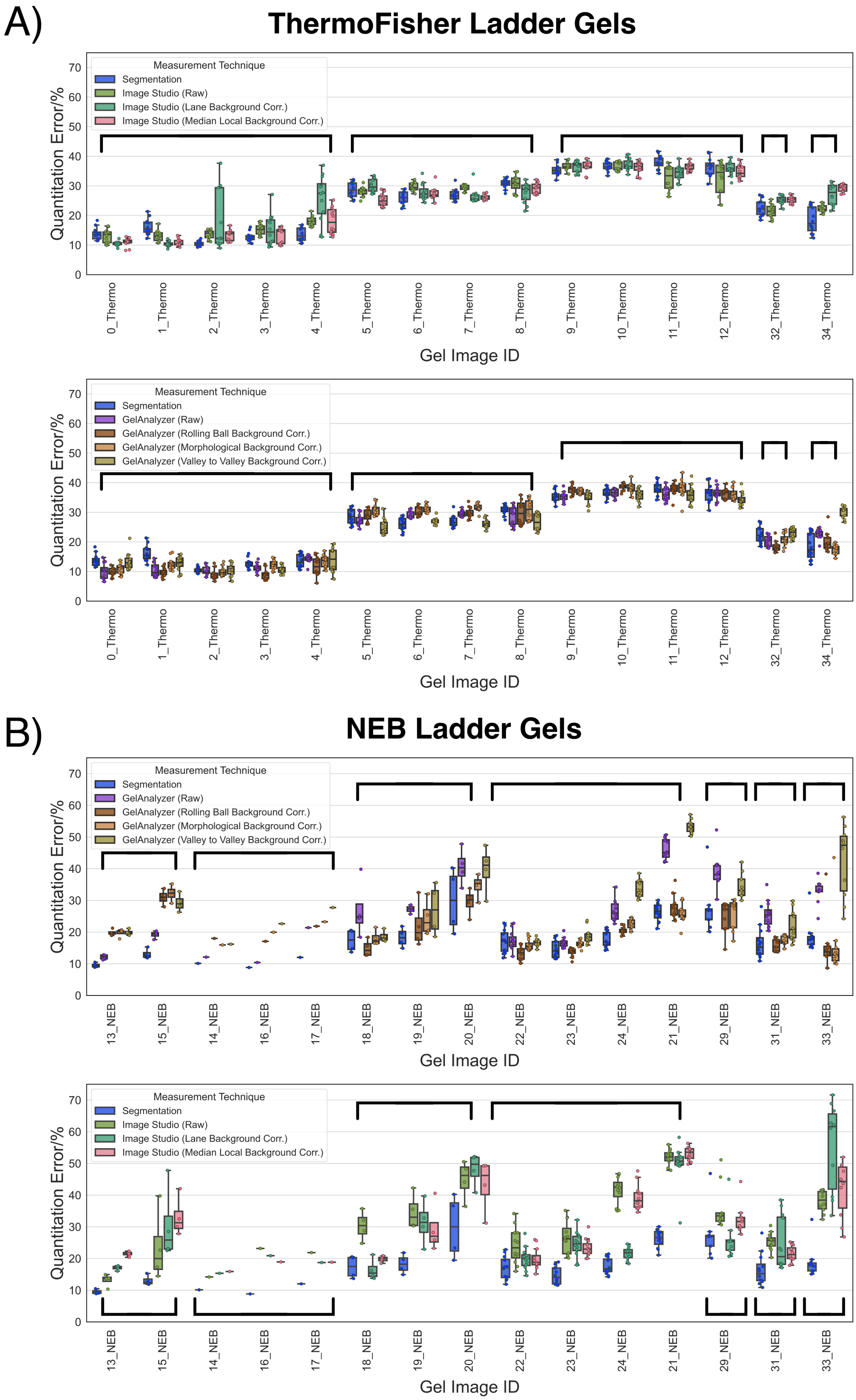

Supp. Fig. 4: The data from Supp. Fig. 3 was rearranged into boxplots split by both ladder (**A)** for ThermoFisher and **B)** for NEB) and individual gel source image. The braces indicate which images came from the same source gel; each image in a group was taken with a different exposure time, with the time increasing from left to right. As demonstrated in Supp. Fig. 2, the data clearly showed that experimental conditions had the largest impact on quantification accuracy. The various background correction methods used have a positive impact in some cases, but also have a negative impact in others (in particular in the NEB dataset). It is clear that no single background correction can be applied across the board for every image type, adding another layer of complexity for conventional lane-based analysis systems. All plots display the median as a horizontal line within each box. The bounds of each box are defined by the lower quartile (25th percentile) and upper quartile (75th percentile). The whiskers extend to the most extreme data points within 1.5 times the interquartile range (IQR) from the quartiles. All individual points for each plot have been overlaid over each sample column.

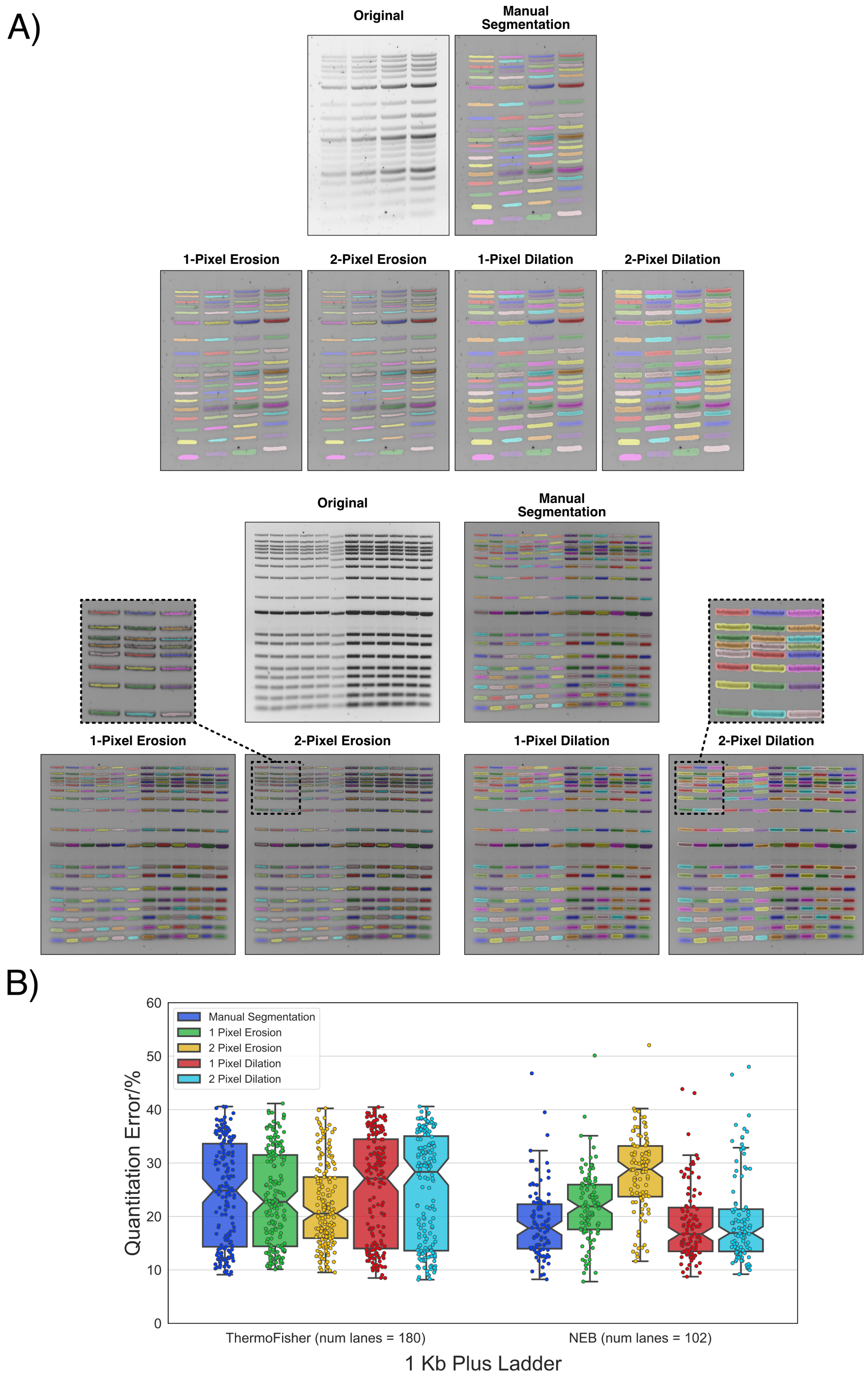

Supp. Fig. 5: Additional erosion/dilation was conducted on the same dataset as analysed in Fig. 1. **A)** Examples from the quantitation dataset showing the effect of erosion and dilation. Band masks with a 2-pixel dilation or erosion were significantly degraded and it is highly unlikely that a trained user would accept segmentation maps of this low quality. Nevertheless, even in the worst case, only a 10% increase in error is expected on average, as is shown in **B)** and Supp. Table 1. For **B)**, the box plots display the median as a horizontal line within each box. The bounds of each box are defined by the lower quartile (25th percentile) and upper quartile (75th percentile). The notches provide the 95% confidence interval around the median. The whiskers extend to the most extreme data points within 1.5 times the interquartile range (IQR) from the quartiles. All individual points for each plot have been overlaid over each box.

Supp. Table. 1: The mean and standard deviation of the error percentages resulting from all lane quantitation analyses conducted on the ladder dataset (including those of Supp. Fig. 5 and Supp. Fig. 3). The best and second-best result for each ladder dataset is marked in red and blue, respectively. The segmentation approaches consistently result in the best possible scores across both datasets, with some variability dependent on pixel labelling subjectivity (as demonstrated in Supp. Fig. 5).

**Method**	**ThermoFisher**		**NEB**	
	Mean	STD	Mean	STD
Segmentation	24.43	9.73	18.82	6.77
Rolling Ball Background Corrected Segmentation	26.19	9.43	19.22	6.09
Patch (Global) Background Corrected Segmentation	27.00	8.88	19.44	7.14
Local Background Corrected Segmentation	29.19	5.95	27.67	8.44
1 Pixel Eroded Segmentation	23.22	8.88	21.93	6.84
2 Pixel Eroded Segmentation	21.93	7.70	28.35	7.39
1 Pixel Dilated Segmentation	24.96	10.18	18.66	6.99
2 Pixel Dilated Segmentation	24.97	10.52	19.60	8.49
GelAnalyzer (Raw)	23.90	10.27	27.61	10.98
GelAnalyzer (Rolling Ball Background Corr.)	23.80	11.57	19.55	6.83
GelAnalyzer (Morphological Background Corr.)	24.74	10.88	21.00	6.54
GelAnalyzer (Valley to Valley Background Corr.)	24.03	9.53	30.13	12.77
Image Studio (Raw)	24.89	8.69	33.18	11.34
Image Studio (Lane Background Corr.)	25.92	9.40	30.48	14.64
Image Studio (Median Local Background Corr.)	24.88	9.37	31.79	12.21

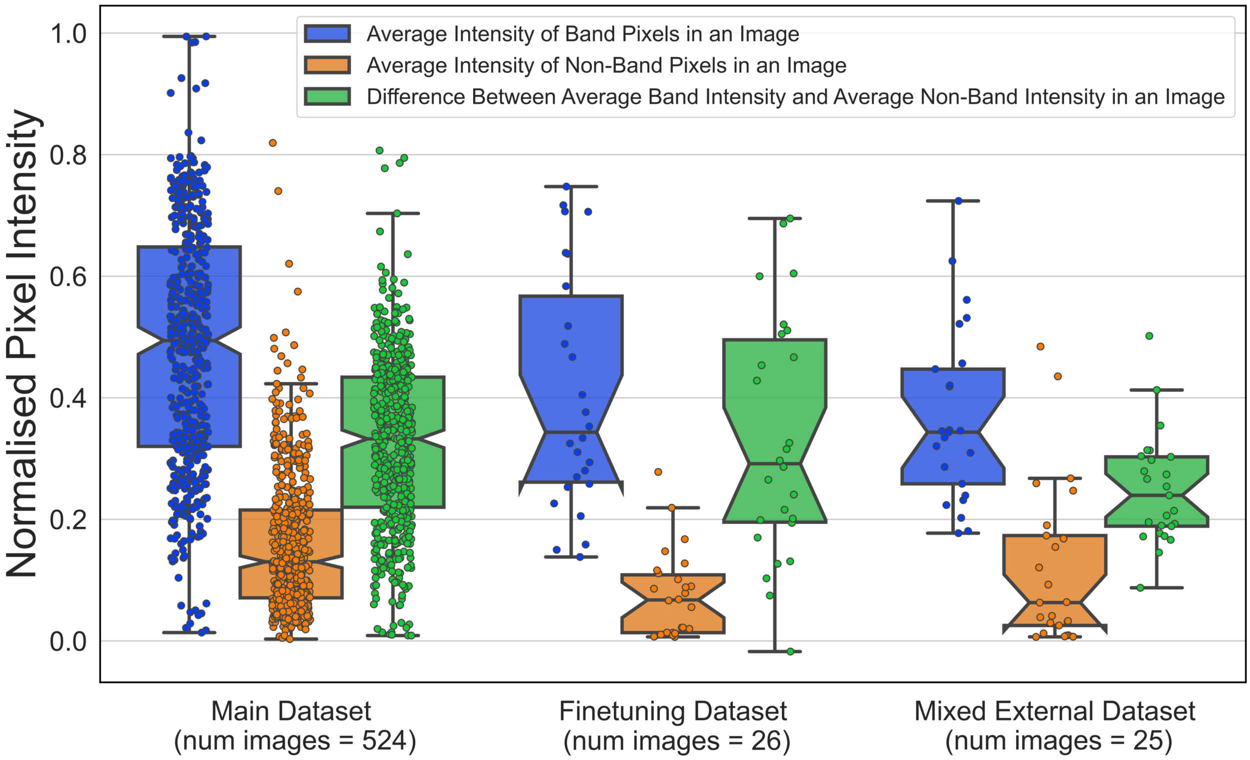

Supp. Fig. 6: Distribution of foreground/background pixel values in the three datasets utilised in this work. The foreground value for each image was calculated by averaging all the pixels within the ground-truth band segmentation masks, and vice-versa for the background value. The main dataset clearly exhibits a highly diverse distribution of foreground/background values. The fine-tuning/external datasets cover a smaller portion of the available space, with the fine-tuning dataset noticeably having lower background levels overall. No average image background intensity exceeds the corresponding average foreground intensity, except for one instance in the fine-tuning dataset. The box plots display the median as a horizontal line within each box. The bounds of each box are defined by the lower quartile (25th percentile) and upper quartile (75th percentile). The notches provide the 95% confidence interval around the median. The whiskers extend to the most extreme data points within 1.5 times the interquartile range (IQR) from the quartiles. All individual points for each plot have been overlaid over each box.

Metric Definitions for Segmentation Analysis (applicable for all subsequent tables and figures)

- **Dice Score:** A standard segmentation metric that compares the similarity of two segmentation maps (the query map and a ground truth map). In this binary prediction task, the Dice Score is equal to the F1 Score. The value is bounded between 0 and 1 (higher is better). The exact formulation is as follows:

$$\text{Dice Score} = \frac{2\,TP}{2\,TP + FP + FN}$$

  Where TP is the number of true positive pixels i.e. pixels correctly predicted as a gel band, FP is the number of false positive pixels, FN is the number of false negative pixels and TN is the number of true negative pixels (not used here).

- **Precision:** A metric that represents the accuracy of positive (i.e. gel band) pixel predictions. The value is bounded between 0 and 1 (higher is better). The exact formulation is as follows:

$$\text{Precision} = \frac{TP}{TP + FP}$$

- **Recall:** A metric that measures how many of the positive pixels were correctly identified by a model. The value is bounded between 0 and 1 (higher is better). The exact formulation is as follows:

$$\text{Recall} = \frac{TP}{TP + FN}$$

- **Hausdorff Distance:** Another standard segmentation metric that measures the worst-case distance between the outlines of two segmentation maps. The lowest possible value is 0 (perfect match), but the highest value is unbounded. We used *Scipy*'s implementation to measure the Hausdorff distance, but in theory this would be formulated as follows:

$$HD(A, B) = \max\left(\sup_{a\in A}\inf_{b\in B} d(a, b), \sup_{b\in B}\inf_{a\in A} d(b, a)\right)$$

  Where $HD(A, B)$ is the Hausdorff Distance between two input segmentation maps A and B, $\sup$ is the supremum operator, $\inf$ is the infimum operator, $d(a, b)$ is the Euclidean distance between two pixels, and a/b are the pixels at the boundaries of each segmentation map.

- **Band Accuracy:** Band-level accuracy is measured by computing the fraction of predicted positive pixels that correctly matched those in the ground truth segmentation map for a particular band. The value is bounded between 0 and 1 (higher is better). This is the only metric that is applied at a per-band level i.e. a distinct value is obtained for each band in an image.

Supp. Table. 2: Quantitative results on the 54-image test set comparing the various U-Net models trained. The best and second-best values for each score are marked in red and blue, respectively. The Dice score and Band Accuracy columns correspond to the same results presented in Fig. 3B, while the full metric distributions for all methods are also provided in Supp. Fig. 8. The Band Accuracy column is the result of individually quantifying the accuracy of all bands (2622) in the test set, while the other scores are all computed once per image (54). The Hausdorff distance (less is better), is unbounded and can become infinite when a segmentation map has no positively identified foreground pixels. This happened in only one case for the LSDB-only U-Net, and this value was ignored from the mean/STD computation. The results clearly show a massive improvement between conventional and machine-learning techniques across the board. The fine-tuned U-Net shows an almost negligible drop in performance compared to the original model across all metrics.

**Model/Method**	**Dice Score**		**Precision**		**Recall**		**Hausdorff Distance**		**Band Accuracy**	
	Mean	STD	Mean	STD	Mean	STD	Mean	STD	Mean	STD
Custom U-Net	0.82	0.13	0.84	0.15	0.84	0.13	118.53	195.13	0.74	0.35
Custom U-Net (fine-tuned)	0.80	0.17	0.86	0.14	0.79	0.17	131.59	208.90	0.74	0.35
nnU-Net	0.83	0.16	0.89	0.10	0.81	0.17	121.17	188.00	0.78	0.32
LSDB-only U-Net	0.21	0.26	0.17	0.28	0.64	0.33	390.64	297.18	0.10	0.26
Multi-Otsu	0.51	0.22	0.58	0.30	0.57	0.24	455.93	294.71	0.23	0.37
Watershed	0.51	0.22	0.65	0.30	0.52	0.24	456.71	294.74	0.27	0.39

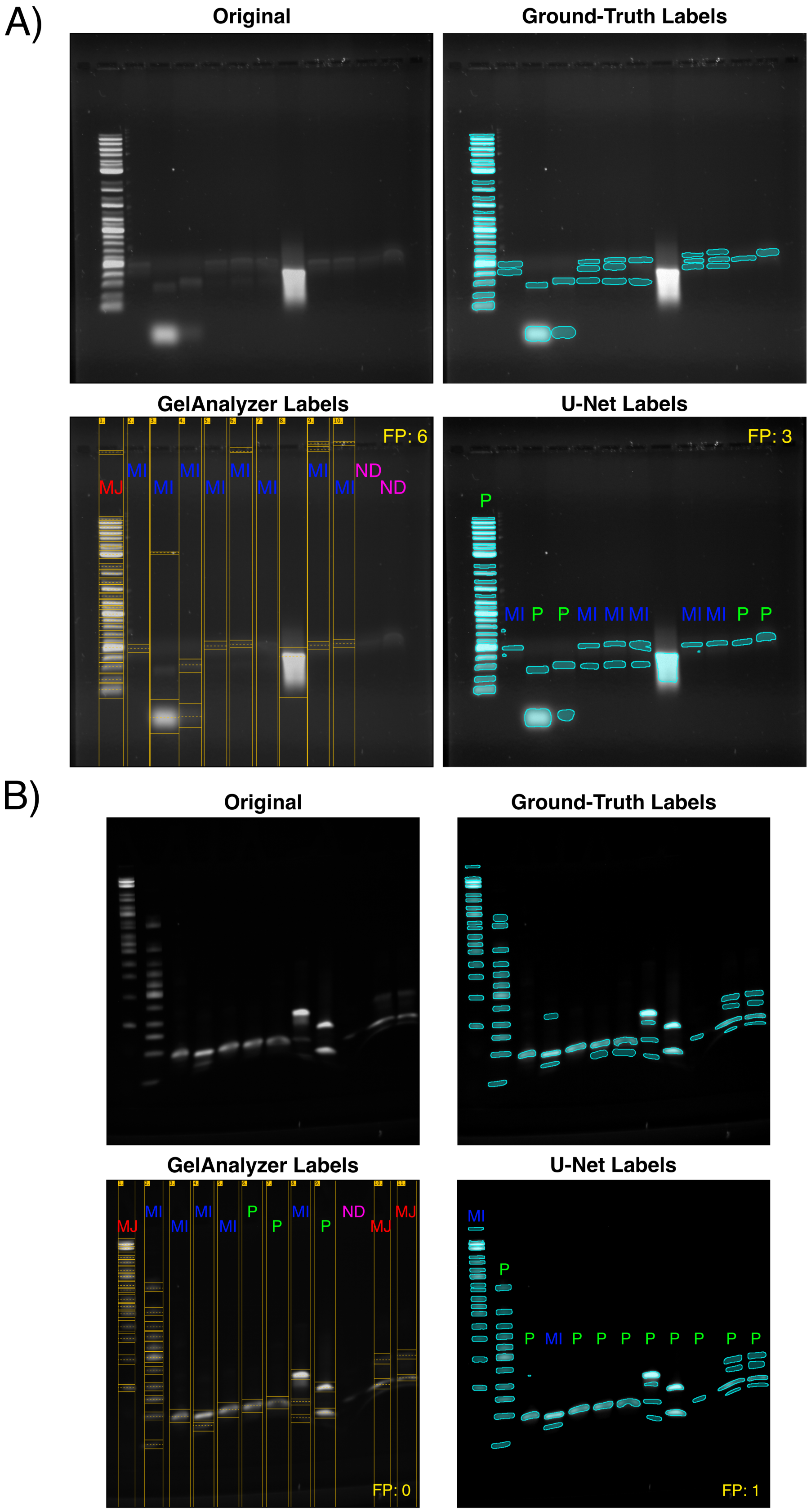

Supp. Fig. 7: Two examples of the qualitative analysis conducted on the test set (Fig. 3D). The GelAnalyzer lanes and bands were generated using the default settings. The U-Net segmentation maps were obtained directly from the model without adjustment. For all the test images, each lane was classified as either: 'minor adjustments required' (MI), 'major adjustments required' (MJ), 'lane missed' (ND) or 'precise bands' (P). A lane was only classified as requiring minor adjustments if just a single band needed to be tweaked to achieve accurate coverage. The false positive count (FP) indicates the number of lanes that have at least one additional incorrect band included.

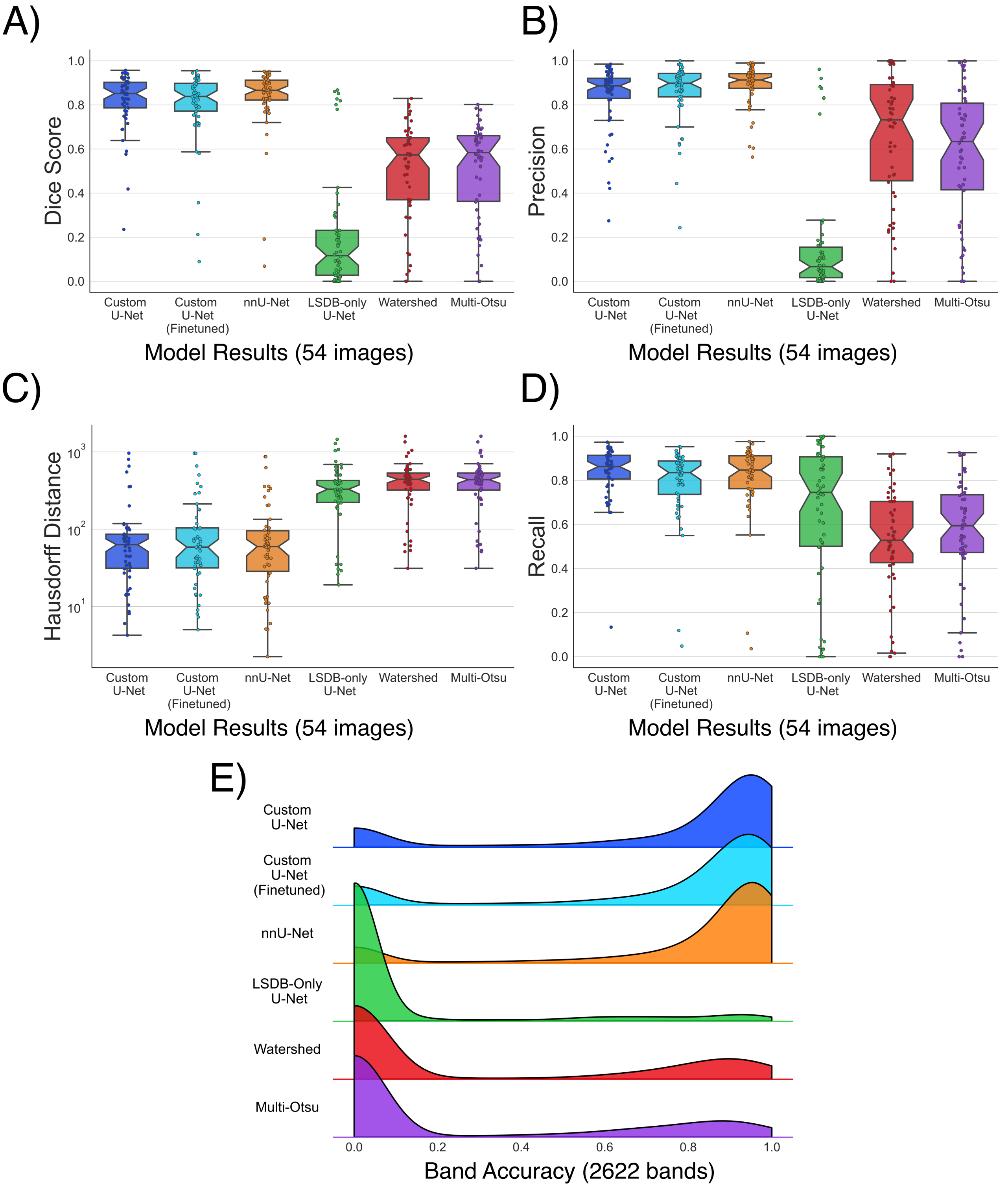

Supp. Fig. 8: Plots showing the overall performance of the models on the test set using various metrics: **A)** Dice Score (higher is better), **B)** Precision (higher is better), **C)** Hausdorff Distance (lower is better), **D)** Recall (higher is better) and **E)** Band Detection Accuracy (higher is better). The Band Accuracy plot is the result of individually quantifying the accuracy of all bands (2622) in the test set, while the other scores are all computed once per image (54). The Hausdorff distance is unbounded and can become infinite when a segmentation map has no positively identified foreground pixels. This happened in only one case for the LSDB-only U-Net, and this value is not displayed in the plot. As in Supp. Table 2, the metrics all show a clear gap between the conventional and machine-learning based techniques. The fine-tuned model shows almost identical performance to the original U-Net, with very minor performance drops across most metrics. For **A)**-**D)**, the box plots display the median as a horizontal line within each box. The bounds of each box are defined by the lower quartile (25th percentile) and upper quartile (75th percentile). The notches provide the 95% confidence interval around the median. The whiskers extend to the most extreme data points within 1.5 times the interquartile range (IQR) from the quartiles. All individual points for each plot have been overlaid over each box.

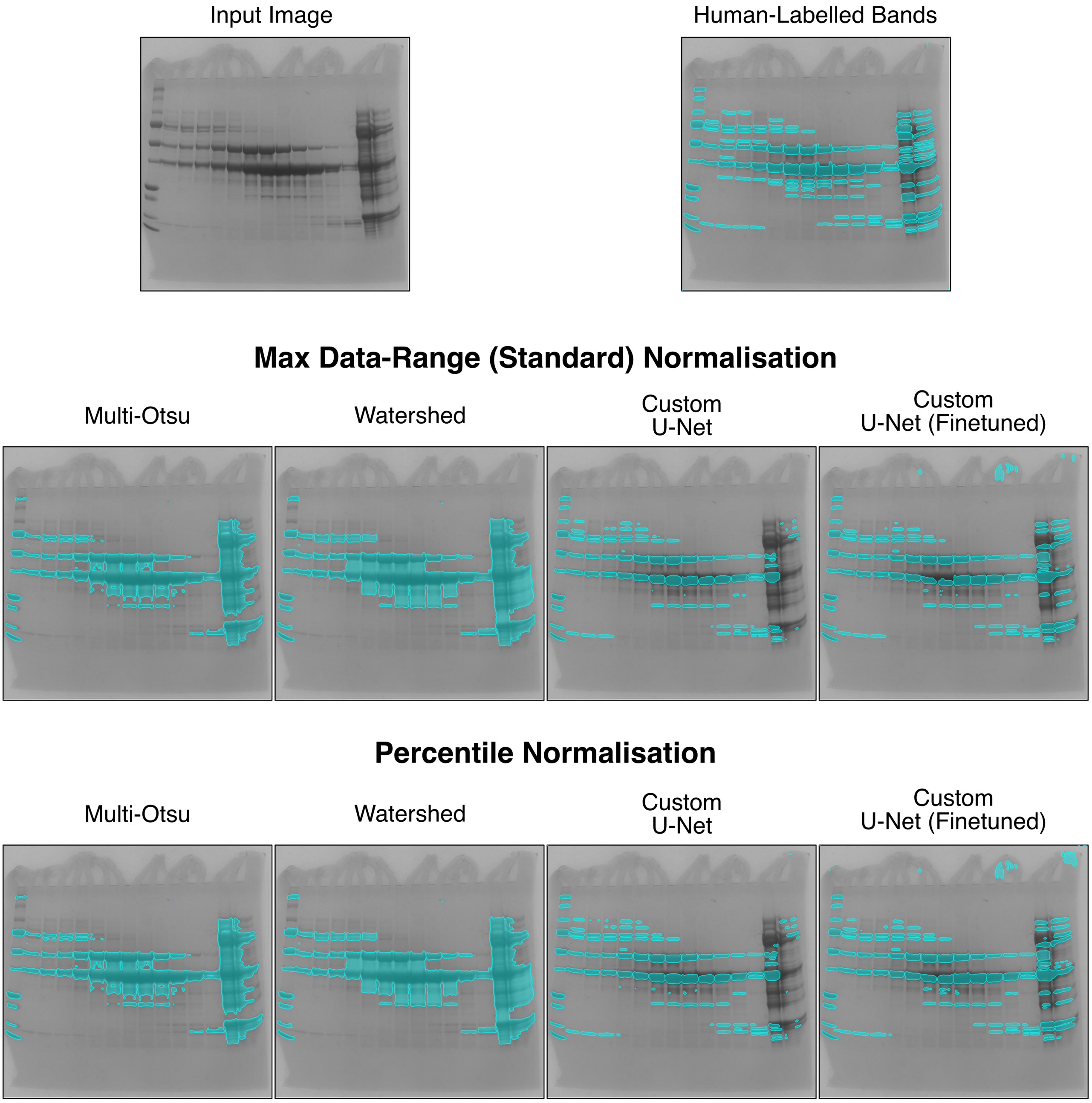

Supp. Fig. 9: Example segmentation maps generated for a PAGE image from the external unseen dataset. The middle row maps were generated after the image was normalised by dividing by the maximum bit-type value (255 for 8-bit and 65535 for 16-bit). The bottom row maps were generated after the image was normalised by percentile normalisation (removing the bottom/top 0.1% and then normalising remainder to 0-1 range). Both U-Net models produced satisfactory results, but the fine-tuned model was capable of identifying more bands at the expense of more false positives at the gel edges.

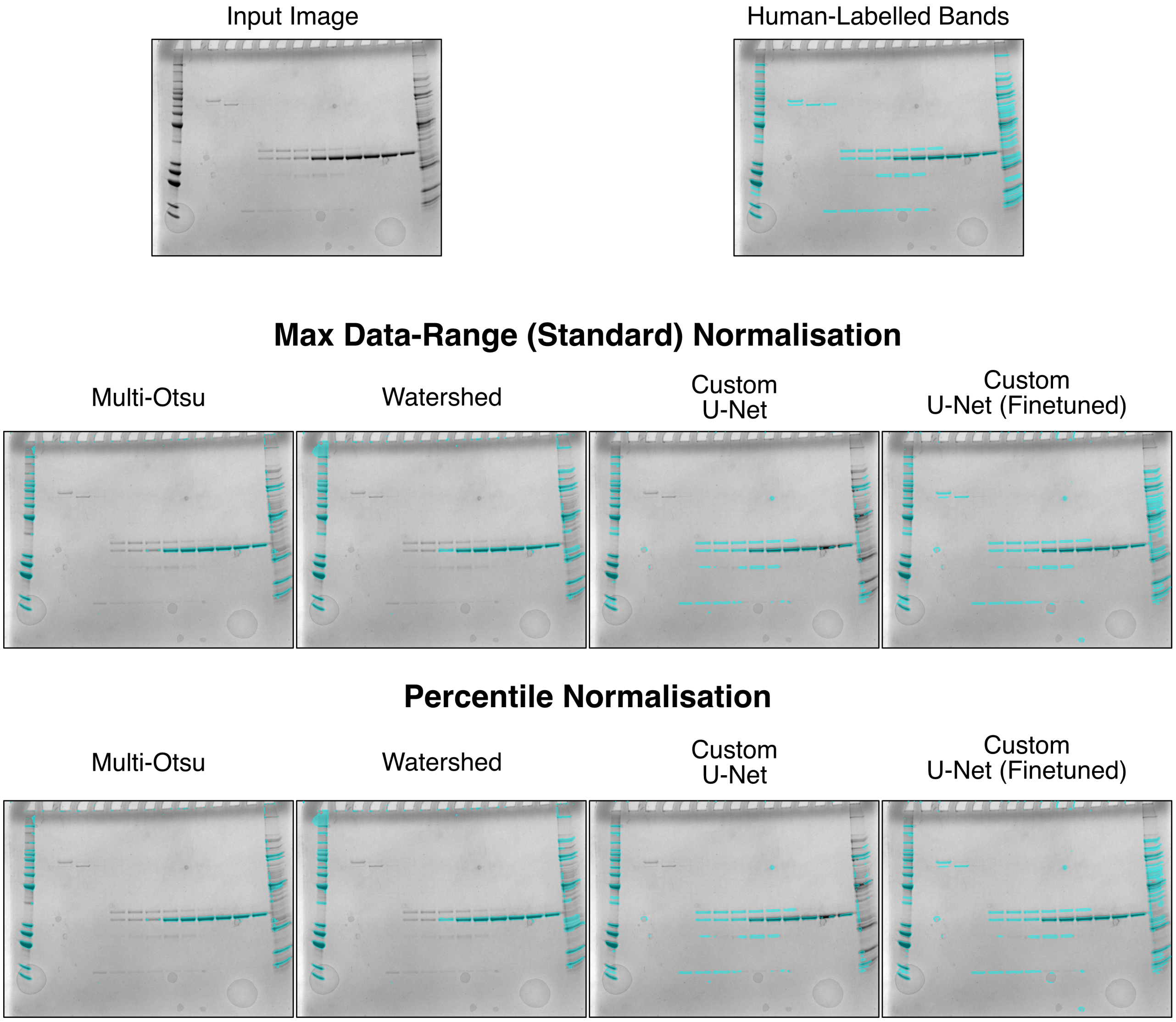

Supp. Fig. 10: Example segmentation maps generated for a PAGE image from the external unseen dataset. The middle row maps were generated after the image was normalised by dividing by the maximum bit-type value (255 for 8-bit and 65535 for 16-bit). The bottom row maps were generated after the image was normalised by percentile normalisation (removing the bottom/top 0.1% and then normalising remainder to 0-1 range). Both U-Net models produced satisfactory results, but the fine-tuned model was capable of identifying more bands at the expense of more false positives at the gel edges.

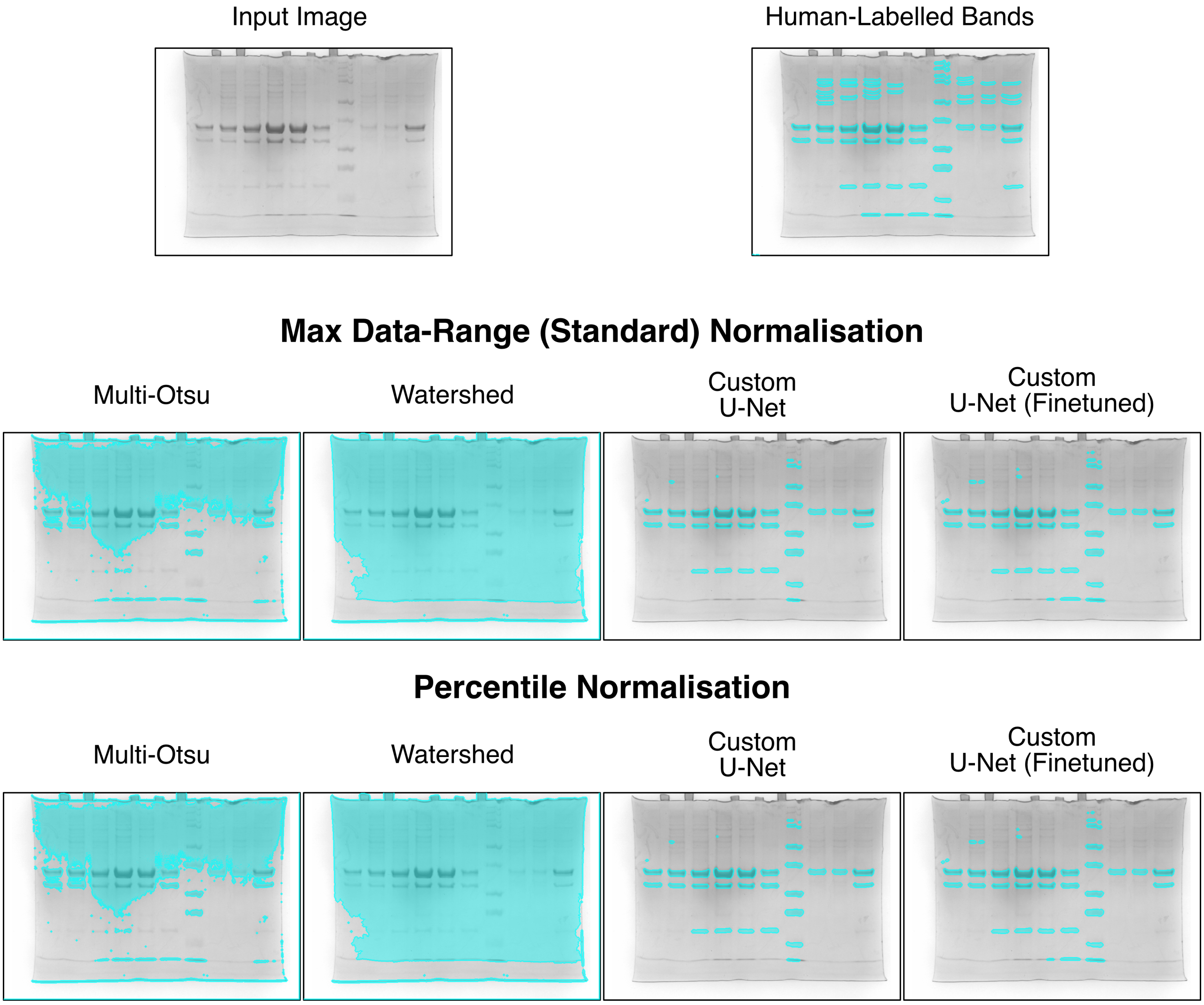

Supp. Fig. 11: Example segmentation maps generated for a PAGE image from the external unseen dataset. The middle row maps were generated after the image was normalised by dividing by the maximum bit-type value (255 for 8-bit and 65535 for 16-bit). The bottom row maps were generated after the image was normalised by percentile normalisation (removing the bottom/top 0.1% and then normalising remainder to 0-1 range). In this case the two U-Nets produced very similar segmentation maps.

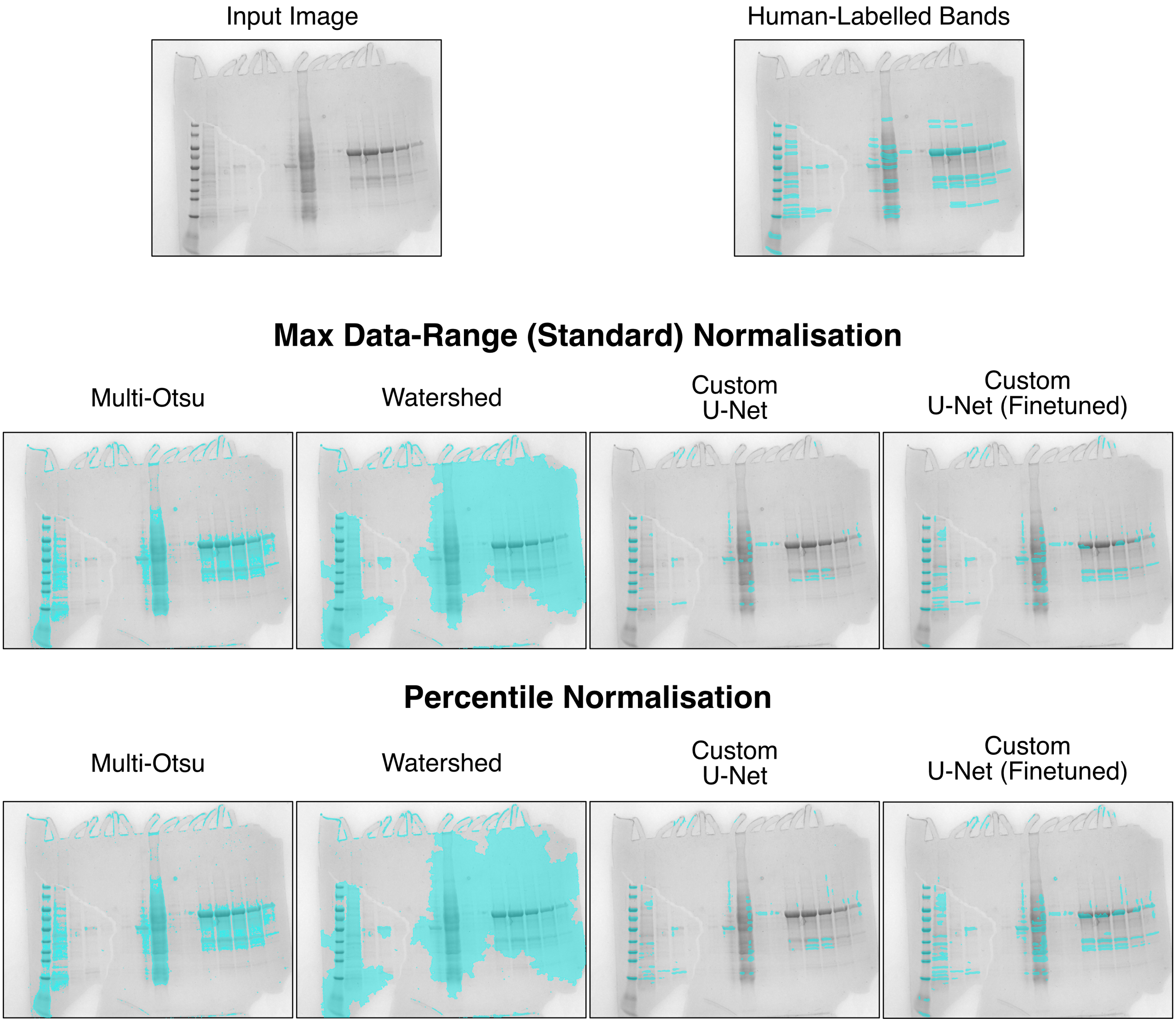

Supp. Fig. 12: Example segmentation maps generated for a PAGE image from the external unseen dataset. The middle row maps were generated after the image was normalised by dividing by the maximum bit-type value (255 for 8-bit and 65535 for 16-bit). The bottom row maps were generated after the image was normalised by percentile normalisation (removing the bottom/top 0.1% and then normalising remainder to 0-1 range). In this case, the standard U-Net model results are weak, but the fine-tuned model was able to restore performance on most well-defined bands. As before, percentile normalisation helped improve band coverage in most cases.

Supp. Table. 3: Quantitative results on the 25-image external unseen dataset comparing the standard and fine-tuned U-Net models. Images were normalised using the max bit-range before segmentation. The best and second-best values for each score are marked in red and blue, respectively. The Band Accuracy column is the result of individually quantifying the accuracy of all bands (1385) in the dataset, while the other scores are all computed once per image (25). The Hausdorff distance (less is better) is unbounded and can become infinite when a segmentation map has no positively identified foreground pixels. This happened in only one case for the Custom U-Net, and this value was ignored from the mean/STD computation. The results continue to show that the U-Net models exhibit superior performance over the conventional algorithms, with the fine-tuned model being the superior option in most cases. However, the average scores are noticeably degraded when compared to the original test set.

**Model/Method**	**Dice Score**		**Precision**		**Recall**		**Hausdorff Distance**		**Band Accuracy**	
	Mean	STD	Mean	STD	Mean	STD	Mean	STD	Mean	STD
Custom U-Net	0.65	0.29	0.58	0.30	0.88	0.20	527.19	392.31	0.53	0.44
Custom U-Net (fine-tuned)	0.79	0.13	0.76	0.18	0.86	0.11	508.28	384.45	0.66	0.41
Multi-Otsu	0.44	0.18	0.49	0.26	0.63	0.31	616.90	384.08	0.17	0.31
Watershed	0.41	0.21	0.60	0.29	0.56	0.35	618.91	383.07	0.17	0.33

Supp. Table. 4: Quantitative results on the 25-image external unseen dataset comparing the standard and fine-tuned U-Net models. Images were percentile normalised (0.1-99.9% range) before segmentation. The best and second-best values for each score are marked in red and blue, respectively. The Band Accuracy column is the result of individually quantifying the accuracy of all bands (1385) in the dataset, while the other scores are all computed once per image (25). Using percentile normalisation improved the performance of all methods, with the fine-tuned model reaching the same level of performance exhibited on the original test set.

**Model/Method**	**Dice Score**		**Precision**		**Recall**		**Hausdorff Distance**		**Band Accuracy**	
	Mean	STD	Mean	STD	Mean	STD	Mean	STD	Mean	STD
Custom U-Net	0.71	0.23	0.65	0.29	0.90	0.08	518.43	371.04	0.57	0.44
Custom U-Net (fine-tuned)	0.83	0.11	0.85	0.15	0.82	0.12	484.28	311.39	0.71	0.40
Multi-Otsu	0.47	0.16	0.55	0.23	0.60	0.31	634.52	382.67	0.18	0.32
Watershed	0.44	0.21	0.66	0.25	0.52	0.34	636.65	381.55	0.18	0.34

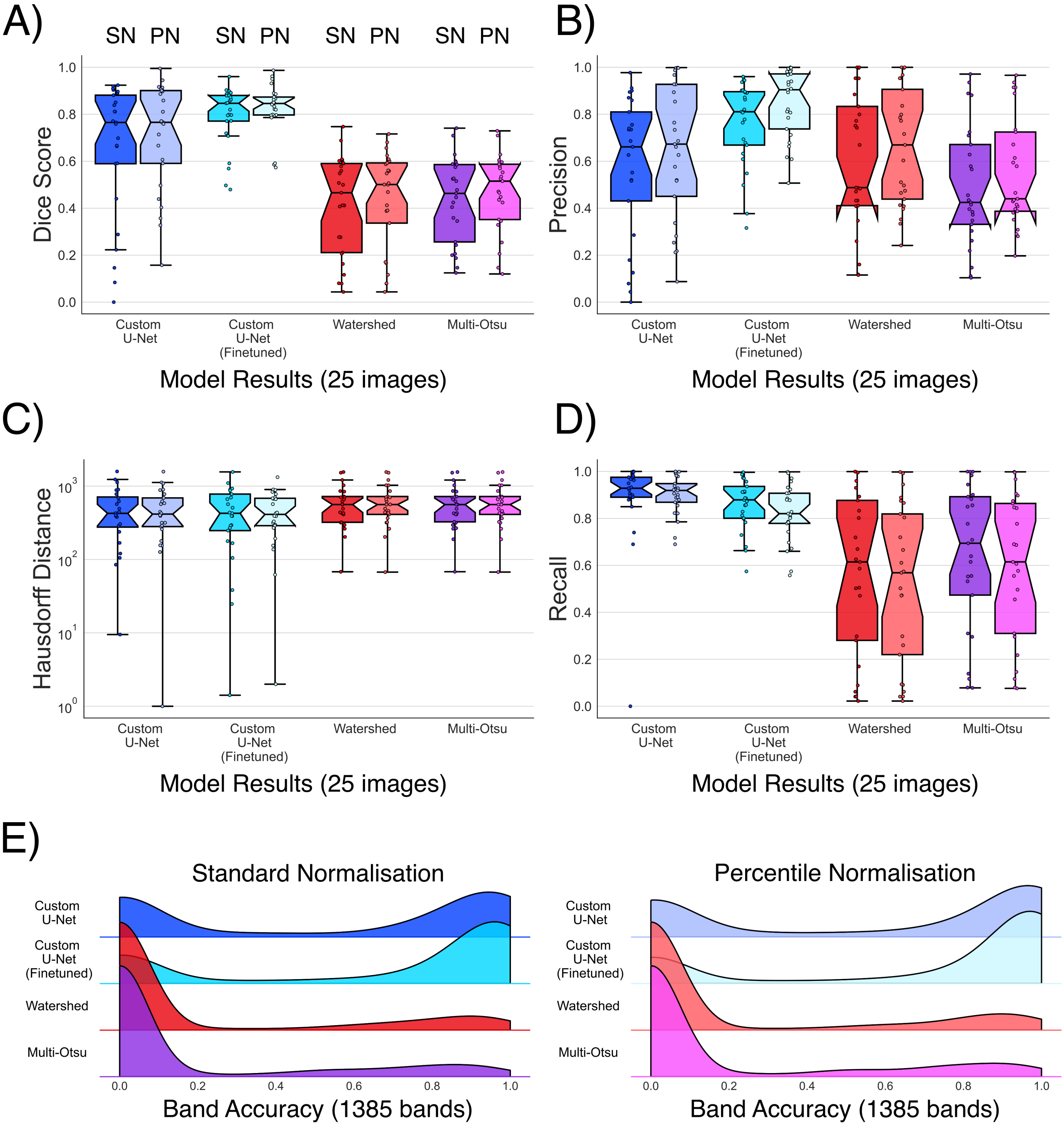

Supp. Fig. 13: Plots showing the overall performance of the custom U-Net models on the external dataset using various metrics: **A)** Dice Score (higher is better), **B)** Precision (higher is better), **C)** Hausdorff Distance (lower is better), **D)** Recall (higher is better) and **E)** Band Detection Accuracy (higher is better). The Band Accuracy plot is the result of individually quantifying the accuracy of all bands (1385) in the test set, while the other scores are all computed once per image (25). For the first 4 plots, each model's standard normalisation (SN) and percentile normalisation (PN) results are plotted in pairs, with the darker shade representing the SN results and lighter shade representing the PN results. For the band accuracy plots, the SN and PN results are separated. The Hausdorff distance is unbounded and can become infinite when a segmentation map has no positively identified foreground pixels. This happened in only one case for the Custom U-Net after standard normalisation, and this value is not displayed in the plot. As in Supp. tables 3 & 4, the metrics all show a clear gap between the conventional and machine-learning based techniques. The fine-tuned model has almost universally superior performance to the standard U-Net across most metrics, except recall. Using percentile normalisation helps improve the performance of both models on the worst images while also slightly improving the best scores. For **A)**-**D)**, the box plots display the median as a horizontal line within each box. The bounds of each box are defined by the lower quartile (25th percentile) and upper quartile (75th percentile). The notches provide the 95% confidence interval around the median. The whiskers extend to the most extreme data points within 1.5 times the interquartile range (IQR) from the quartiles. All individual points for each plot have been overlaid over each box.

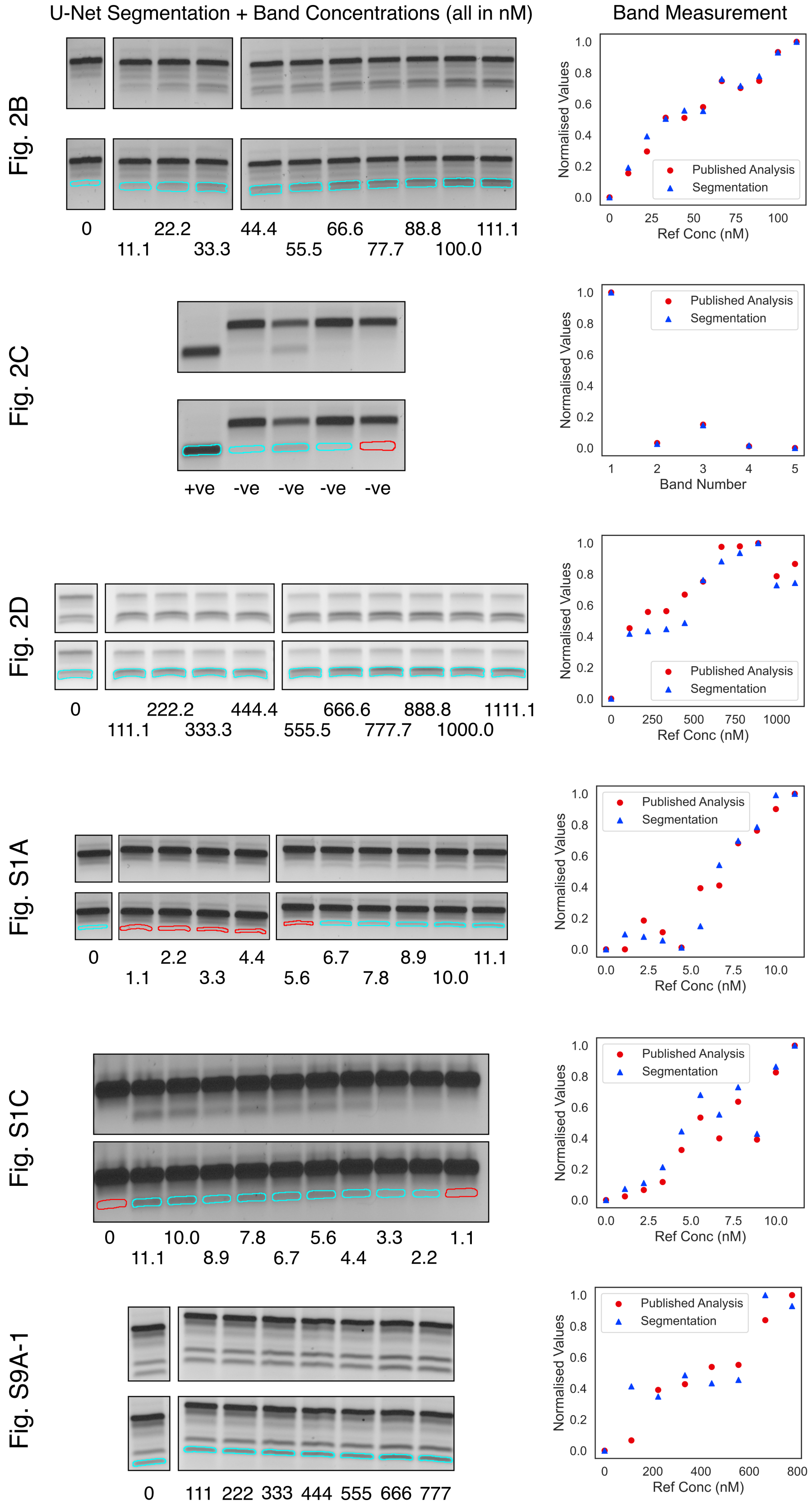

Supp. Fig. 14: Re-analysis of 6 gel images extracted from ‘Multiplexed Label-Free Biomarker Detection by Targeted Disassembly of Variable-Length DNA Payload Chains’ (https://doi.org/10.1002/anse.202200082), which complement the other two analyses provided in Fig. 4A. The left-hand-side of each panel shows the segmentation map generated by the lightweight U-Net, while the right-hand-side shows how the segmentation results compare with the paper’s original analysis. The cyan segmentation regions were generated directly from the U-Net. The red segmentation regions had to be added in manually as they are covering areas where bands are non-existent or too faint to detect.
