# Supplementary material for: GelGenie: an AI-powered framework for gel electrophoresis image analysis
Source: Nat Commun. 2025 May 5;16:4087. doi: 10.1038/s41467-025-59189-0 (PMC12053679; doi:10.1038/s41467-025-59189-0)
Supplement: Supplementary file 2 — Description of Additional Supplementary Information [file 41467_2025_59189_MOESM2_ESM.docx]

**Description of Additional Supplementary Files**

File Name: Supplementary Data 1

Description: Full T-test results (two-sided alternative hypothesis) comparing the results of all methods on the NEB ladder images discussed in Figure 1C.

File Name: Supplementary Data 2

Description: Full T-test results (two-sided alternative hypothesis) comparing the results of all methods on the ThermoFisher ladder images discussed in Figure 1C.

File Name: Supplementary Data 3

Description: Full T-test results (one-sided alternative hypothesis) comparing the results of raw segmentation with the three background correction methods discussed in Figure 1D (NEB ladder images only).

File Name: Supplementary Data 4

Description: Full T-test results (one-sided alternative hypothesis) comparing the results of raw segmentation with the three background correction methods discussed in Figure 1D (ThermoFisher ladder images only).

All results can be reproduced using the code provided at https://github.com/mattaq31/GelGenie/tree/main/pythongelgenie/paper_figure_generation.
