# Supplementary material for: GelGenie: an AI-powered framework for gel electrophoresis image analysis
Source: Nat Commun. 2025 May 5;16:4087. doi: 10.1038/s41467-025-59189-0 (PMC12053679; doi:10.1038/s41467-025-59189-0)
Supplement: Supplementary file 7 — Reporting Summary [file 41467_2025_59189_MOESM7_ESM.pdf]

Corresponding author(s): Matthew Aquilina, Katherine E. Dunn

Last updated by author(s): Mar 18, 2025

Reporting Summary

Nature Portfolio wishes to improve the reproducibility of the work that we publish. This form provides structure for consistency and transparency in reporting. For further information on Nature Portfolio policies, see our Editorial Policies and the Editorial Policy Checklist.

Statistics

For all statistical analyses, confirm that the following items are present in the figure legend, table legend, main text, or Methods section.

n/a	Confirmed	
☐	☒	The exact sample size (*n*) for each experimental group/condition, given as a discrete number and unit of measurement
☐	☒	A statement on whether measurements were taken from distinct samples or whether the same sample was measured repeatedly
☐	☒	The statistical test(s) used AND whether they are one- or two-sided<br>*Only common tests should be described solely by name; describe more complex techniques in the Methods section.*
☒	☐	A description of all covariates tested
☒	☐	A description of any assumptions or corrections, such as tests of normality and adjustment for multiple comparisons
☐	☒	A full description of the statistical parameters including central tendency (e.g. means) or other basic estimates (e.g. regression coefficient) AND variation (e.g. standard deviation) or associated estimates of uncertainty (e.g. confidence intervals)
☐	☒	For null hypothesis testing, the test statistic (e.g. *F*, *t*, *r*) with confidence intervals, effect sizes, degrees of freedom and *P* value noted<br>*Give P values as exact values whenever suitable.*
☒	☐	For Bayesian analysis, information on the choice of priors and Markov chain Monte Carlo settings
☒	☐	For hierarchical and complex designs, identification of the appropriate level for tests and full reporting of outcomes
☒	☐	Estimates of effect sizes (e.g. Cohen's *d*, Pearson's *r*), indicating how they were calculated

Our web collection on statistics for biologists contains articles on many of the points above.

Software and code

Policy information about availability of computer code

Data collection

We created our own custom open-source software (built using Python version 3.10 and various conda-forge packages, listed below) to aid with data collection. All details and the code itself are provided in https://github.com/mattaq31/GelGenie. We also used QuPath (open-source software) version 0.5 to help with image segmentation. We built our graphical GelGenie package as an extension within QuPath itself. We compared our results with GelAnalyzer version 23.1 and LI-COR's ImageStudio version 6.0. The full list of Python packages used is provided below:

setuptools>=59.8.0
scikit-image>=0.19.3
numpy>=1.21.6
pillow>=9.1.1
matplotlib>=3.5.2
imagecodecs>=2021.6.8
scipy>=1.7.3
jsonpickle>=2.2.0
pandas>=1.3.5
aiohttp>=3.8.1
wandb>=0.12.20
tqdm>=4.64.0
imageio>=2.19.3
albumentations>=1.2.1

click>=8.1.3
toml>=0.10.2
torchinfo>=1.7.0
rich>=13.2
rich-click>=1.6.1
opencv>=4.5.1
torchshow>=0.4.2
segmentation-models-pytorch>=0.3.0
monai>=1.2.0

Data analysis: Identical to the above.

For manuscripts utilizing custom algorithms or software that are central to the research but not yet described in published literature, software must be made available to editors and reviewers. We strongly encourage code deposition in a community repository (e.g. GitHub). See the Nature Portfolio guidelines for submitting code & software for further information.

Data

Policy information about availability of data

All manuscripts must include a data availability statement. This statement should provide the following information, where applicable:

- Accession codes, unique identifiers, or web links for publicly available datasets
- A description of any restrictions on data availability
- For clinical datasets or third party data, please ensure that the statement adheres to our policy

The gel datasets generated in this study have been deposited in the publicly-available Zenodo database with DOI 10.5281/zenodo.14641949 (https://doi.org/10.5281/zenodo.14641949). All model weights have also been open-sourced, for which access instructions can be found at https://github.com/mattaq31/GelGenie. Furthermore, the lightweight and finetuned U-Net models have been deposited on BioImage.io with accession IDs `self-disciplined-blowfish' and `trustworthy-llama', respectively.

Supplementary figures and tables have been provided in a separate PDF document. Four supplementary csv files have also been provided, which contain the entire results of the statistical testing of the dataset described in Fig. 1.

Research involving human participants, their data, or biological material

Policy information about studies with human participants or human data. See also policy information about sex, gender (identity/presentation), and sexual orientation and race, ethnicity and racism.

Reporting on sex and gender	We did not conduct human research.
Reporting on race, ethnicity, or other socially relevant groupings	We did not conduct human research.
Population characteristics	We did not conduct human research.
Recruitment	We did not conduct human research.
Ethics oversight	We did not conduct human research.

Note that full information on the approval of the study protocol must also be provided in the manuscript.

Field-specific reporting

Please select the one below that is the best fit for your research. If you are not sure, read the appropriate sections before making your selection.

- [x] Life sciences
- [ ] Behavioural & social sciences
- [ ] Ecological, evolutionary & environmental sciences

For a reference copy of the document with all sections, see nature.com/documents/nr-reporting-summary-flat.pdf

Life sciences study design

All studies must disclose on these points even when the disclosure is negative.

Sample size

For the quantitative t-tests conducted in Figure 1, we generated enough samples to have at least 100 lanes for both commercial ladders analyzed. 100 samples are sufficient for an effect size of 0.3, a power of 80% and a significance level of 0.05. For lower effect sizes, if a difference is present between the methods considered, this will have next to no practical impact given the high error levels inherent to gel electrophoresis quantitation. The full statistical results are provided in the manuscript and the supplementary information.

For Figure 3, the data trends (both qualitative and quantitative) were strong enough that it was deemed unnecessary to increase the sample size of the test set from the 54 images selected. The extended external test set of 25 additional images further confirmed these results.

Data exclusions	We included as many gels as we could to train and analyze the models. Gels were not excluded by any criteria during the dataset preparation. For the quantitation test of Figure 1, all gels were accepted except those that were clearly corrupted by experimental issues (e.g. one gel was run with too high a voltage and half the bands were wiped out). No gels were removed after analysis was started (e.g. due to being outliers).
Replication	The findings were reproduced consistently throughout our unseen test set, as well as with unseen data provided by external sources (Figures 3 and 4).
Randomization	We collected the gel images we used for training and testing from two sources: an open-source online dataset (https://dbarchive.biosciencedbc.jp/en/rgp-caps/data-2.html) and gel images generated from two different members of our laboratory. We collected as many images as we could from both sources. The data was not ordered in any particular way. The data labellers were not given any context on the contents of each image (except for those of Figure 1, where the intent was obvious) - they were simply told to label all bands in each image. For the ladder test of Figure 1, we collected as many gels as we could to reach the target criteria of 100 lanes per commercial ladder.<br><br>The data included in the training/validation/test splits for model training was randomized and care was taken that a proportional random sample from each data source was allocated to each split.
Blinding	The data labellers were not given any context on the contents of each gel image they were labelling (except for the ladder tests of Figure 1).

Reporting for specific materials, systems and methods

We require information from authors about some types of materials, experimental systems and methods used in many studies. Here, indicate whether each material, system or method listed is relevant to your study. If you are not sure if a list item applies to your research, read the appropriate section before selecting a response.

Materials & experimental systems

n/a	Involved in the study
☒	☐ Antibodies
☒	☐ Eukaryotic cell lines
☒	☐ Palaeontology and archaeology
☒	☐ Animals and other organisms
☒	☐ Clinical data
☒	☐ Dual use research of concern
☒	☐ Plants

Methods

n/a	Involved in the study
☒	☐ ChIP-seq
☒	☐ Flow cytometry
☒	☐ MRI-based neuroimaging

Plants

Seed stocks	We did not conduct plant research.
Novel plant genotypes	We did not conduct plant research.
Authentication	We did not conduct plant research.
