# Supplementary figures and images for: GelGenie: an AI-powered framework for gel electrophoresis image analysis
Source: Nat Commun. 2025 May 5;16:4087. doi: 10.1038/s41467-025-59189-0 (PMC12053679; doi:10.1038/s41467-025-59189-0)

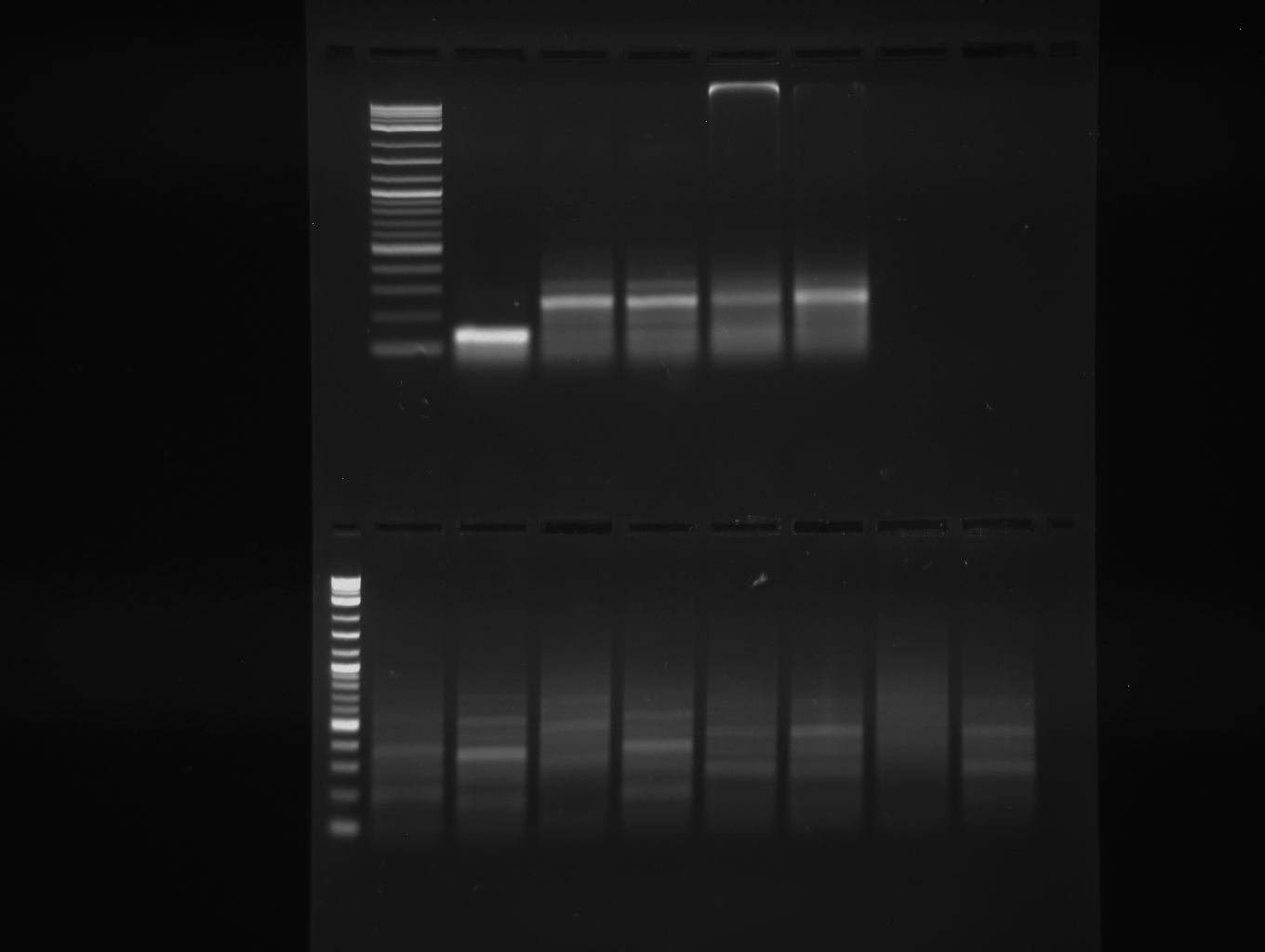

Supplement: Supplementary file 9 — Source Data [file 41467_2025_59189_MOESM9_ESM.zip › source_data/gels/fig_3c_2.tif]

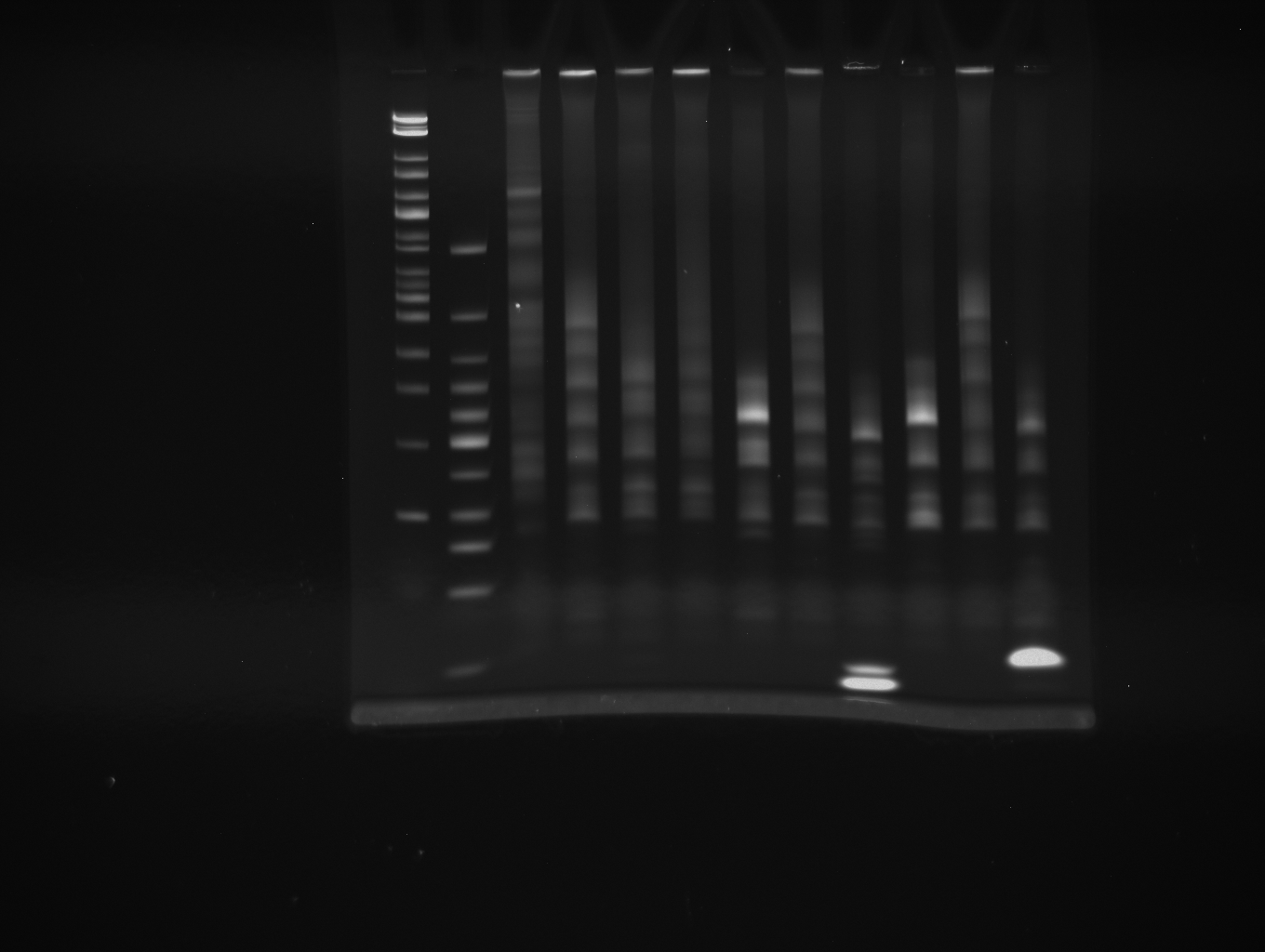

Supplement: Supplementary file 9 — Source Data [file 41467_2025_59189_MOESM9_ESM.zip › source_data/gels/fig_3a_1.tif]

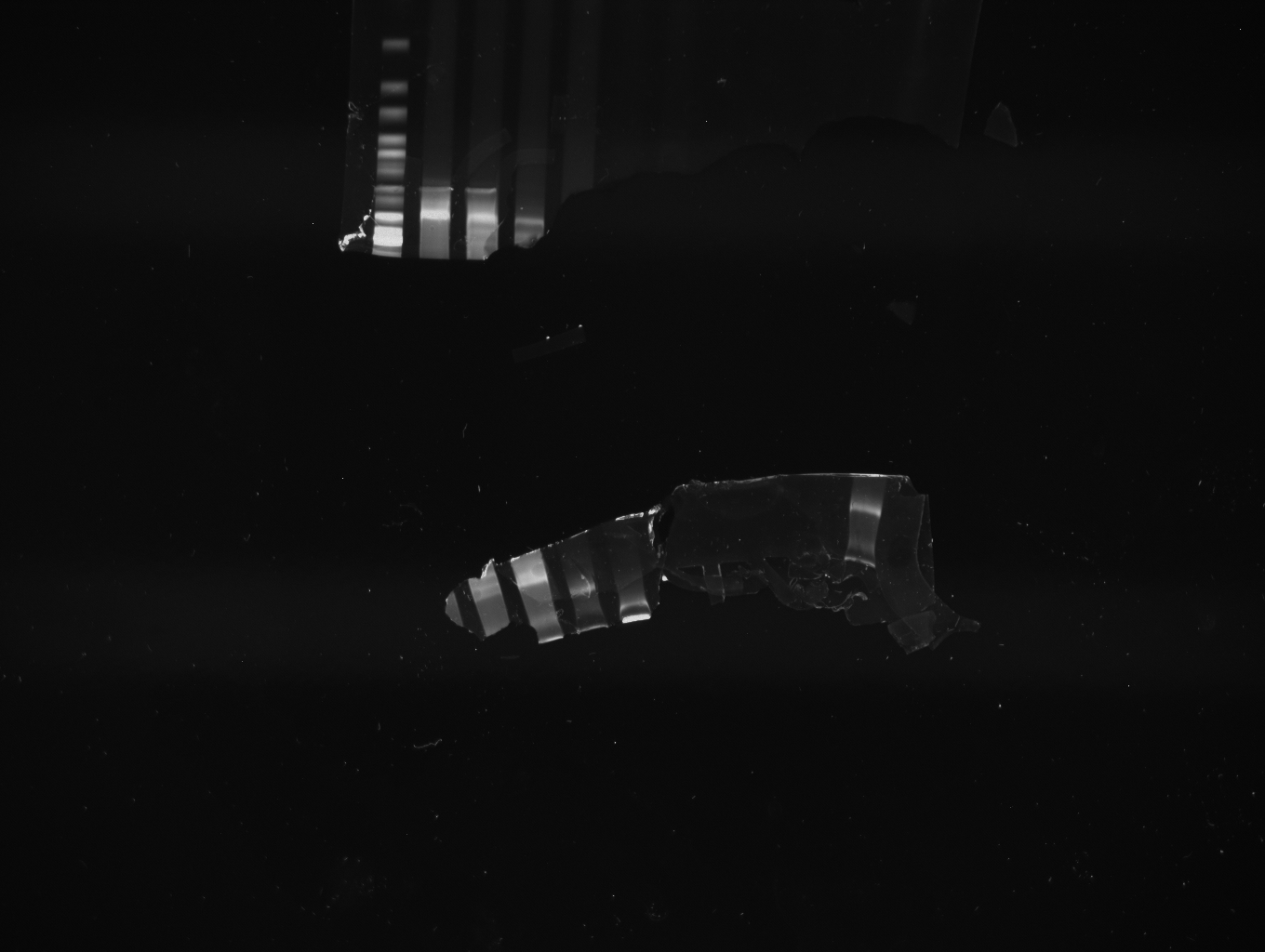

Supplement: Supplementary file 9 — Source Data [file 41467_2025_59189_MOESM9_ESM.zip › source_data/gels/fig_3a_3.tif]

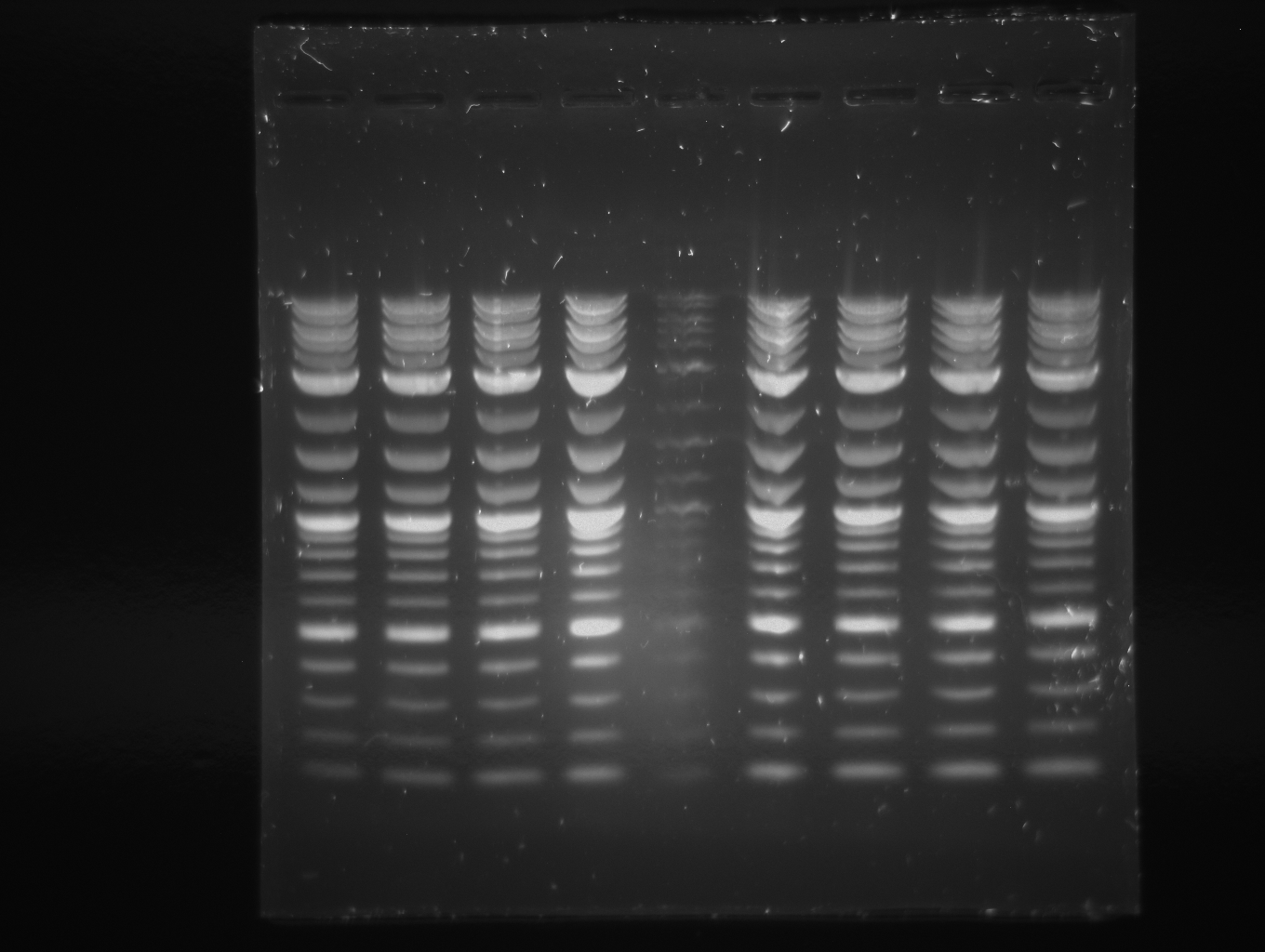

Supplement: Supplementary file 9 — Source Data [file 41467_2025_59189_MOESM9_ESM.zip › source_data/gels/fig_3c_1.tif]

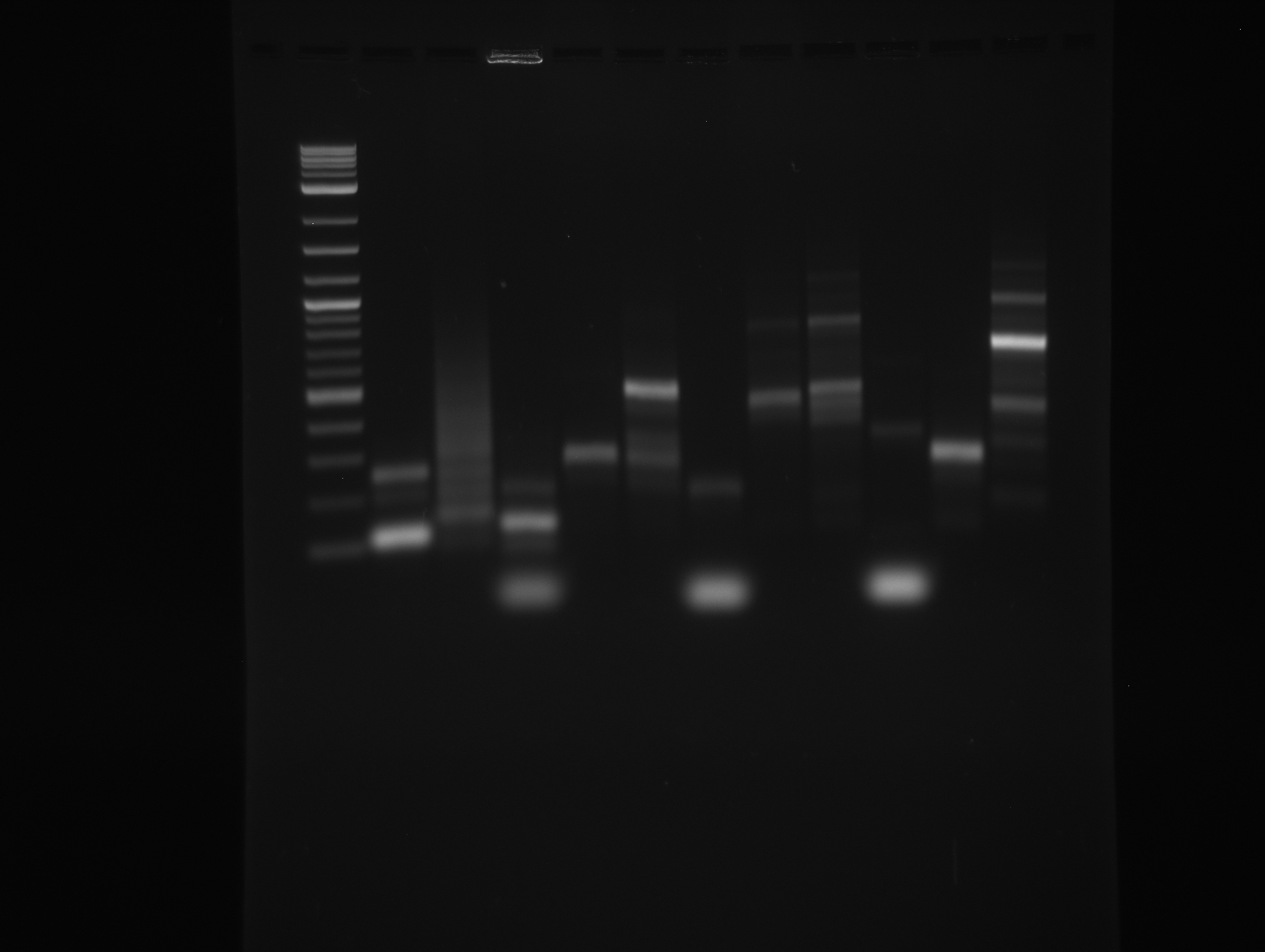

Supplement: Supplementary file 9 — Source Data [file 41467_2025_59189_MOESM9_ESM.zip › source_data/gels/fig_3a_2.tif]

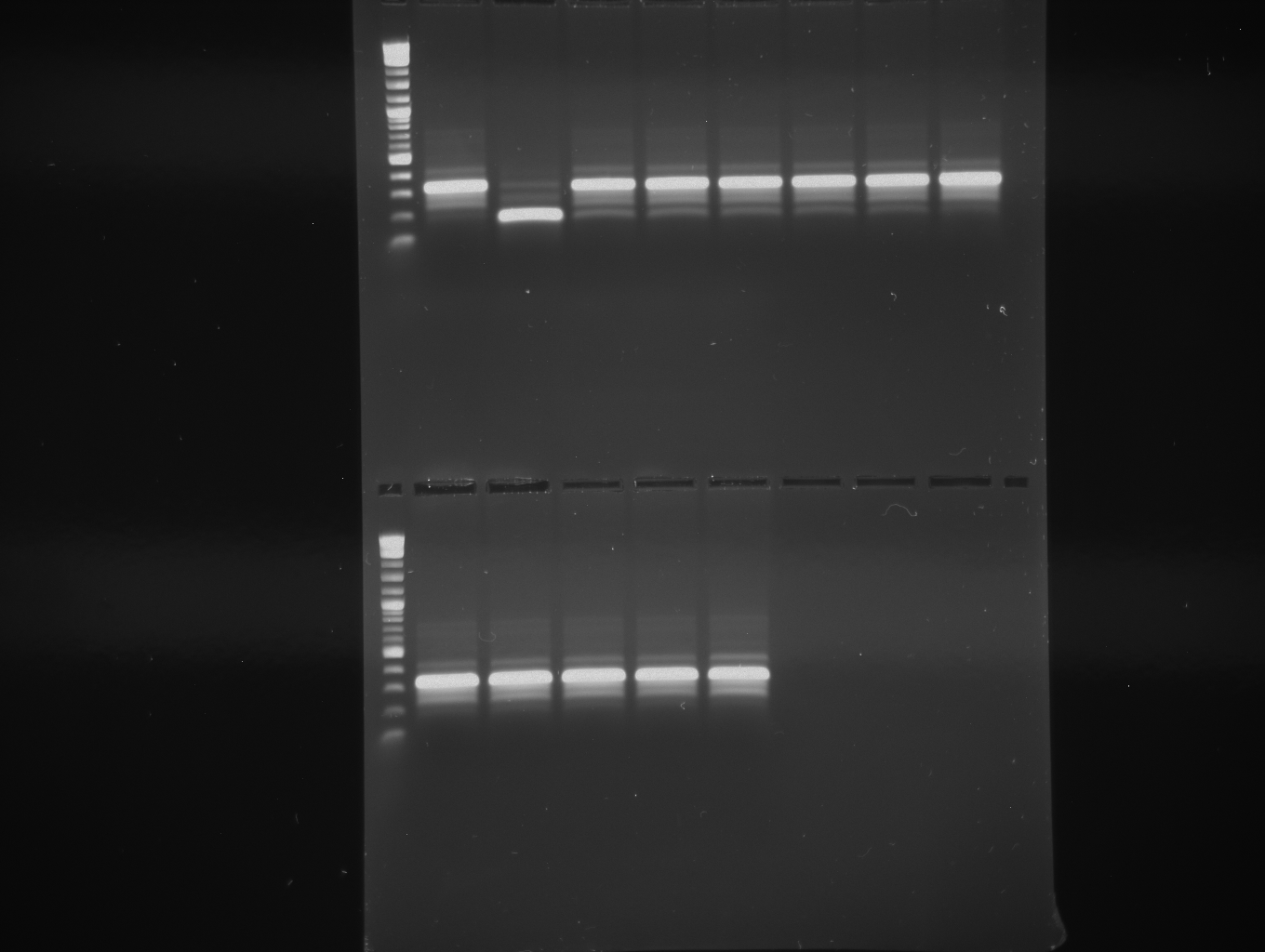

Supplement: Supplementary file 9 — Source Data [file 41467_2025_59189_MOESM9_ESM.zip › source_data/gels/fig_s14_S1A.tif]

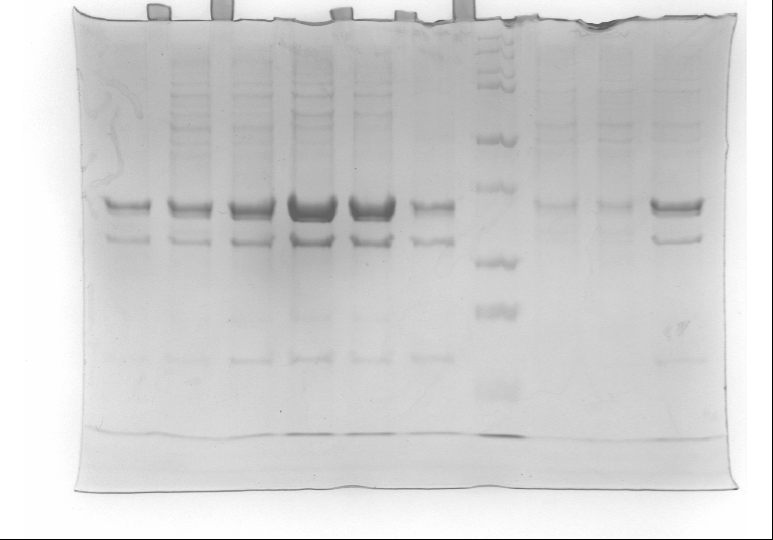

Supplement: Supplementary file 9 — Source Data [file 41467_2025_59189_MOESM9_ESM.zip › source_data/gels/fig_s11.tif]

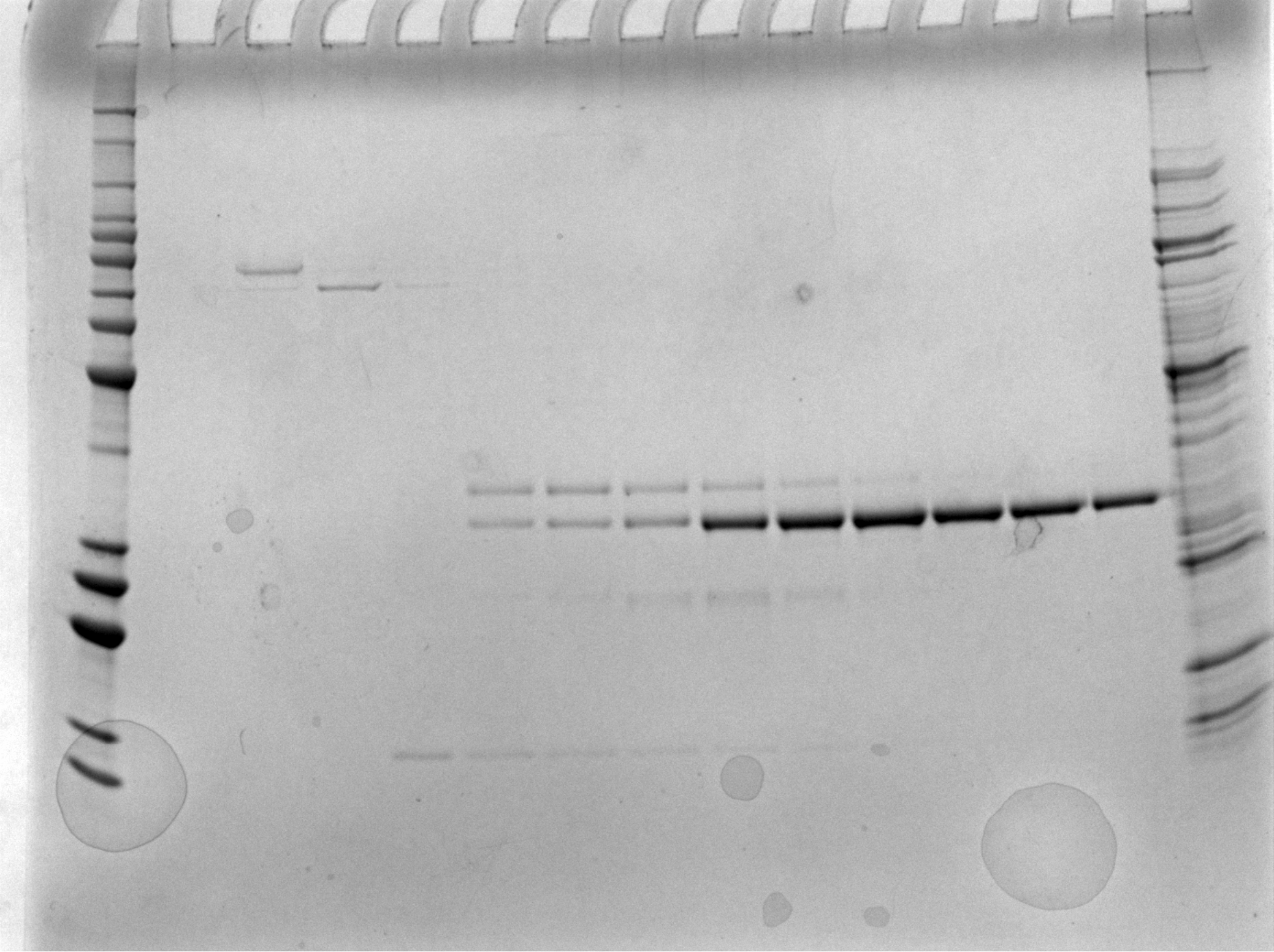

Supplement: Supplementary file 9 — Source Data [file 41467_2025_59189_MOESM9_ESM.zip › source_data/gels/fig_s10.tif]

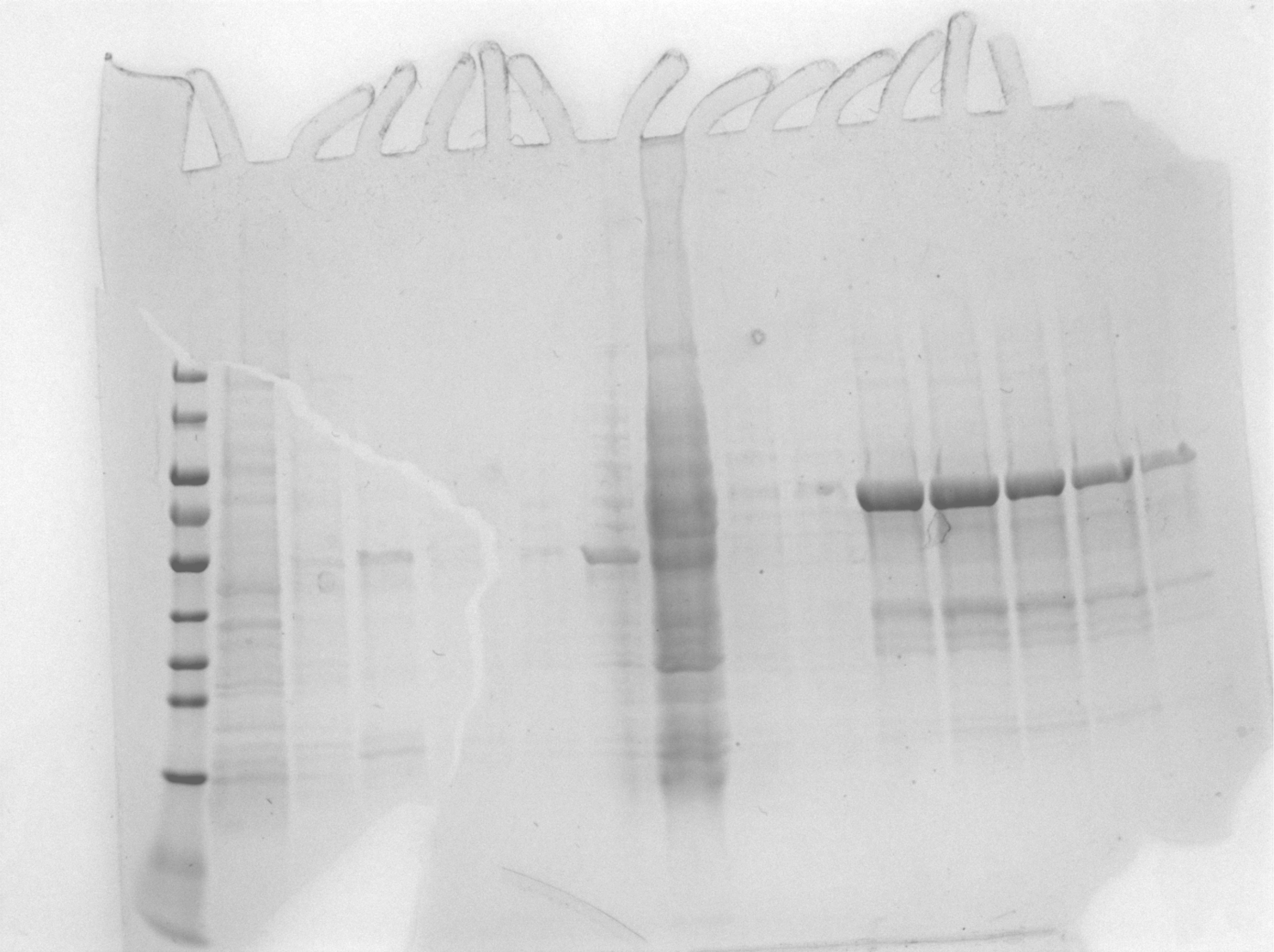

Supplement: Supplementary file 9 — Source Data [file 41467_2025_59189_MOESM9_ESM.zip › source_data/gels/fig_s12.tif]

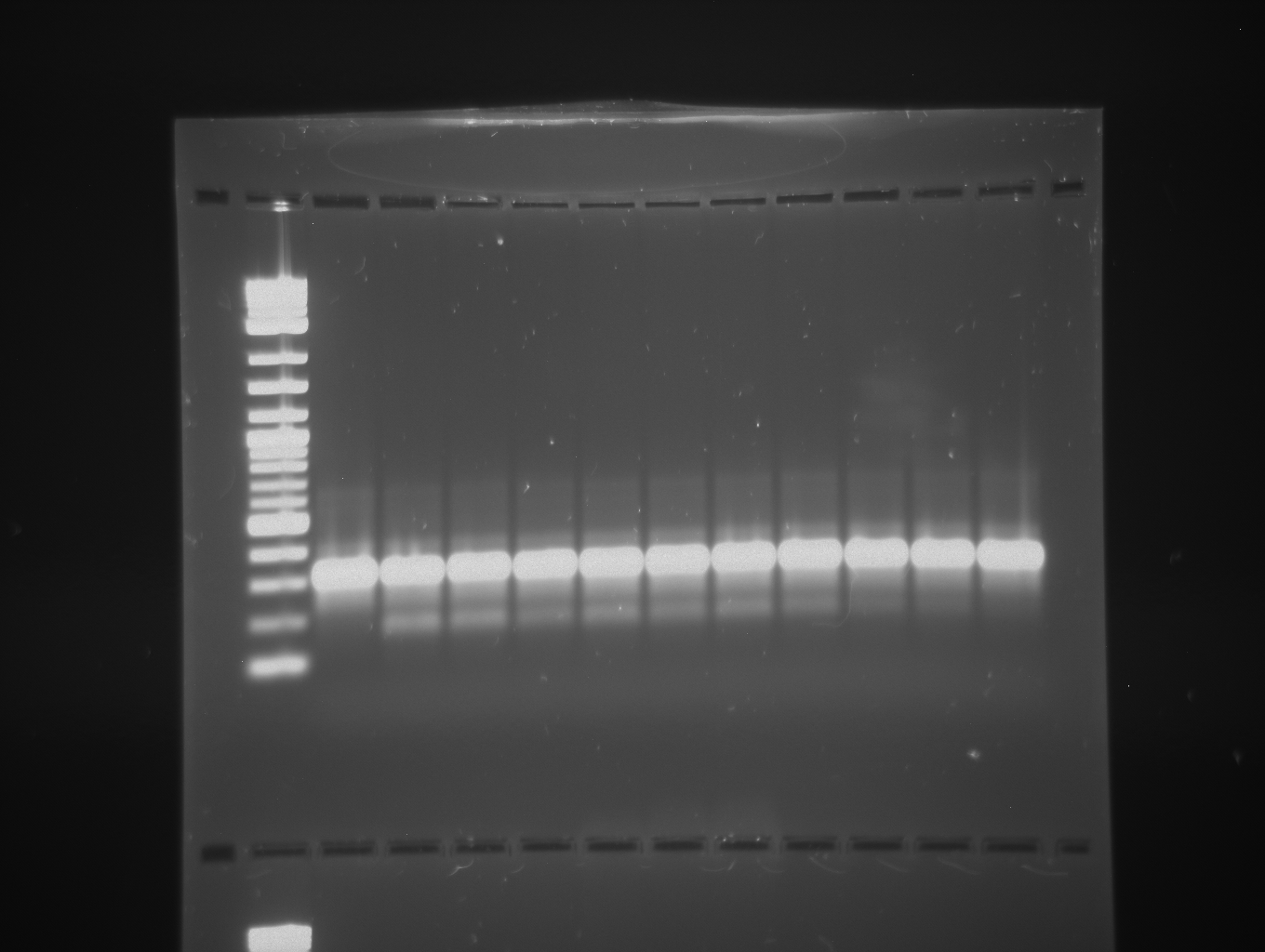

Supplement: Supplementary file 9 — Source Data [file 41467_2025_59189_MOESM9_ESM.zip › source_data/gels/fig_s14_S1C.tif]

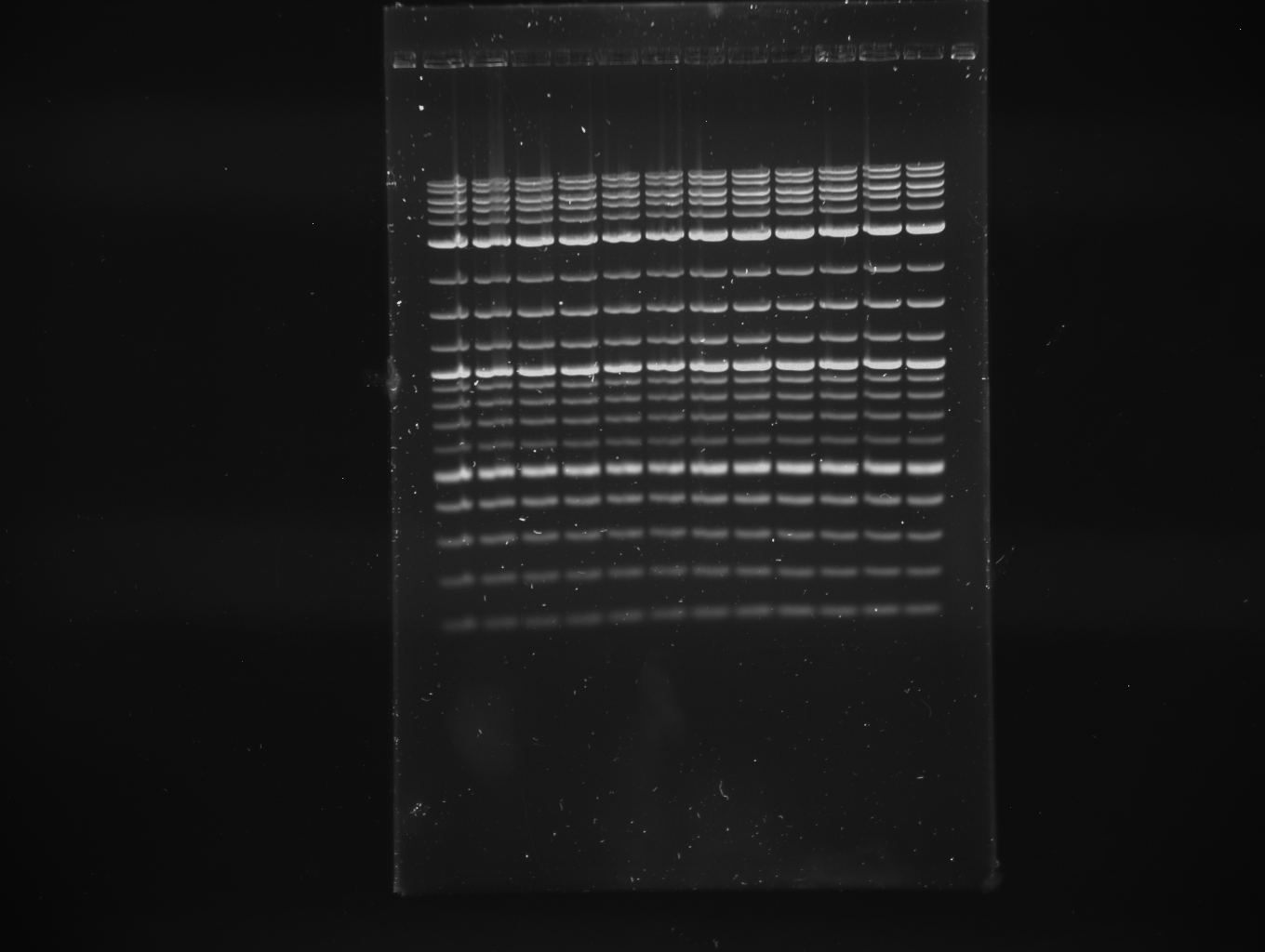

Supplement: Supplementary file 9 — Source Data [file 41467_2025_59189_MOESM9_ESM.zip › source_data/gels/fig_1b.tif]

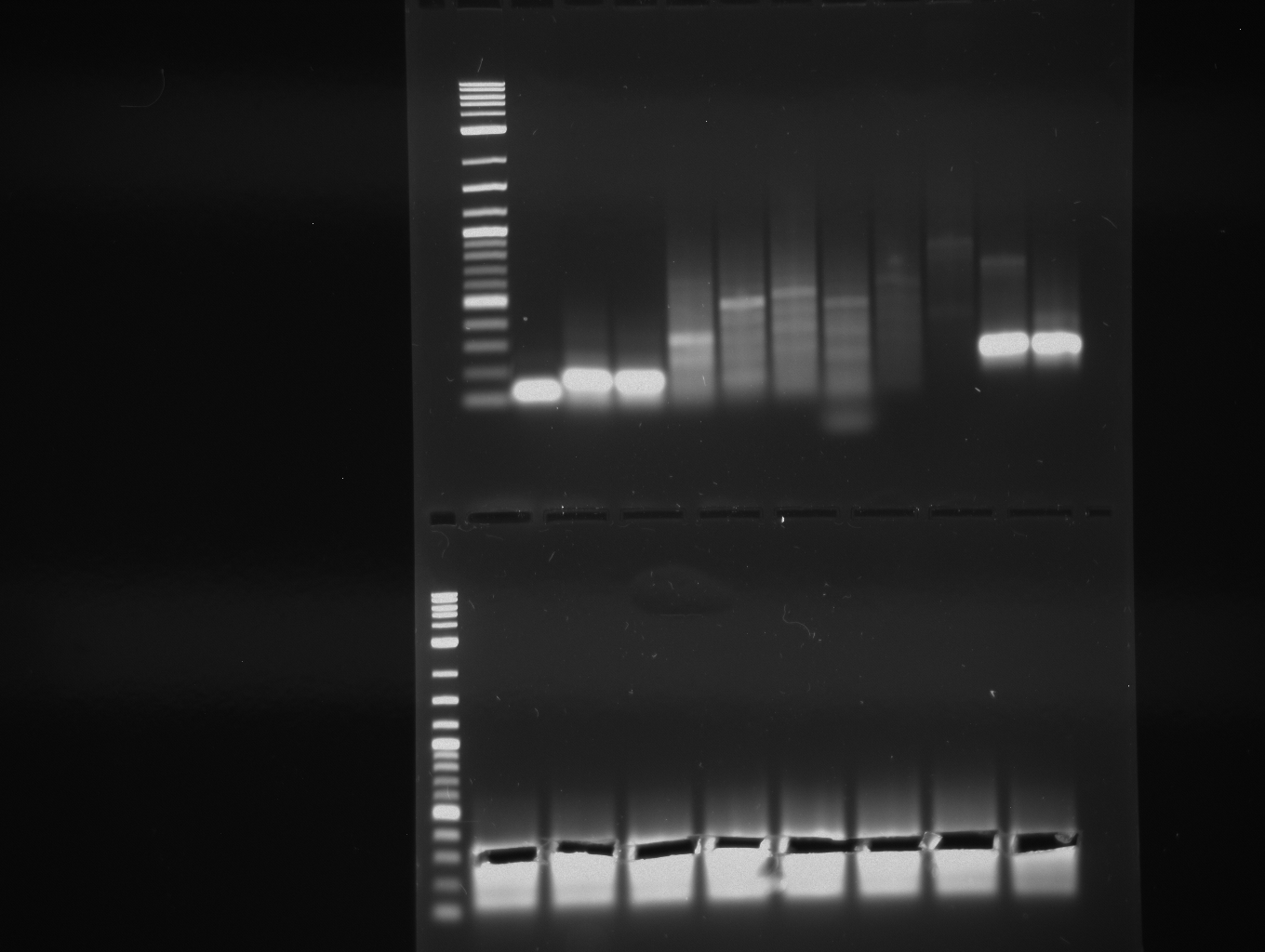

Supplement: Supplementary file 9 — Source Data [file 41467_2025_59189_MOESM9_ESM.zip › source_data/gels/fig_5.tif]

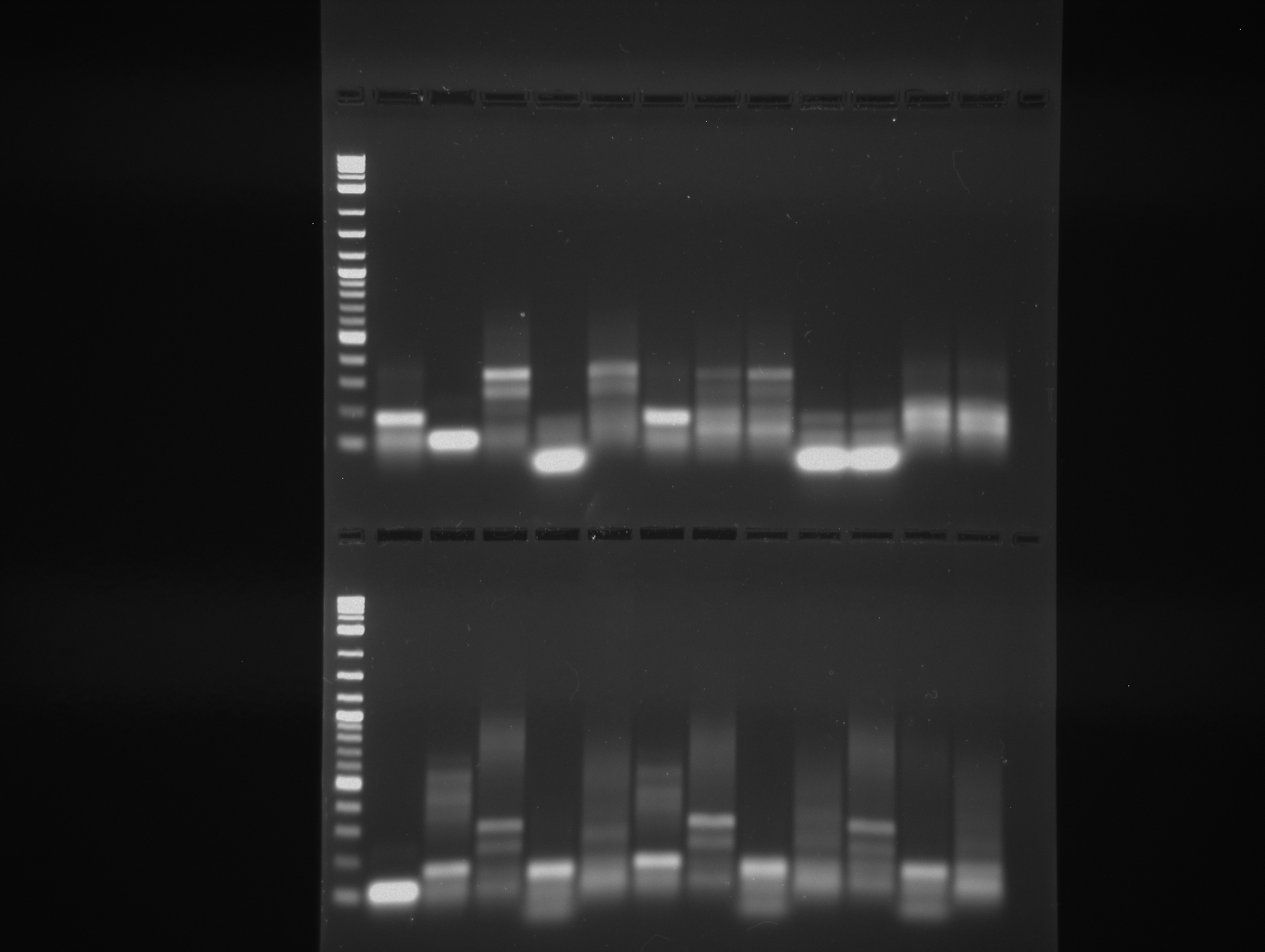

Supplement: Supplementary file 9 — Source Data [file 41467_2025_59189_MOESM9_ESM.zip › source_data/gels/fig_2a_2.tif]

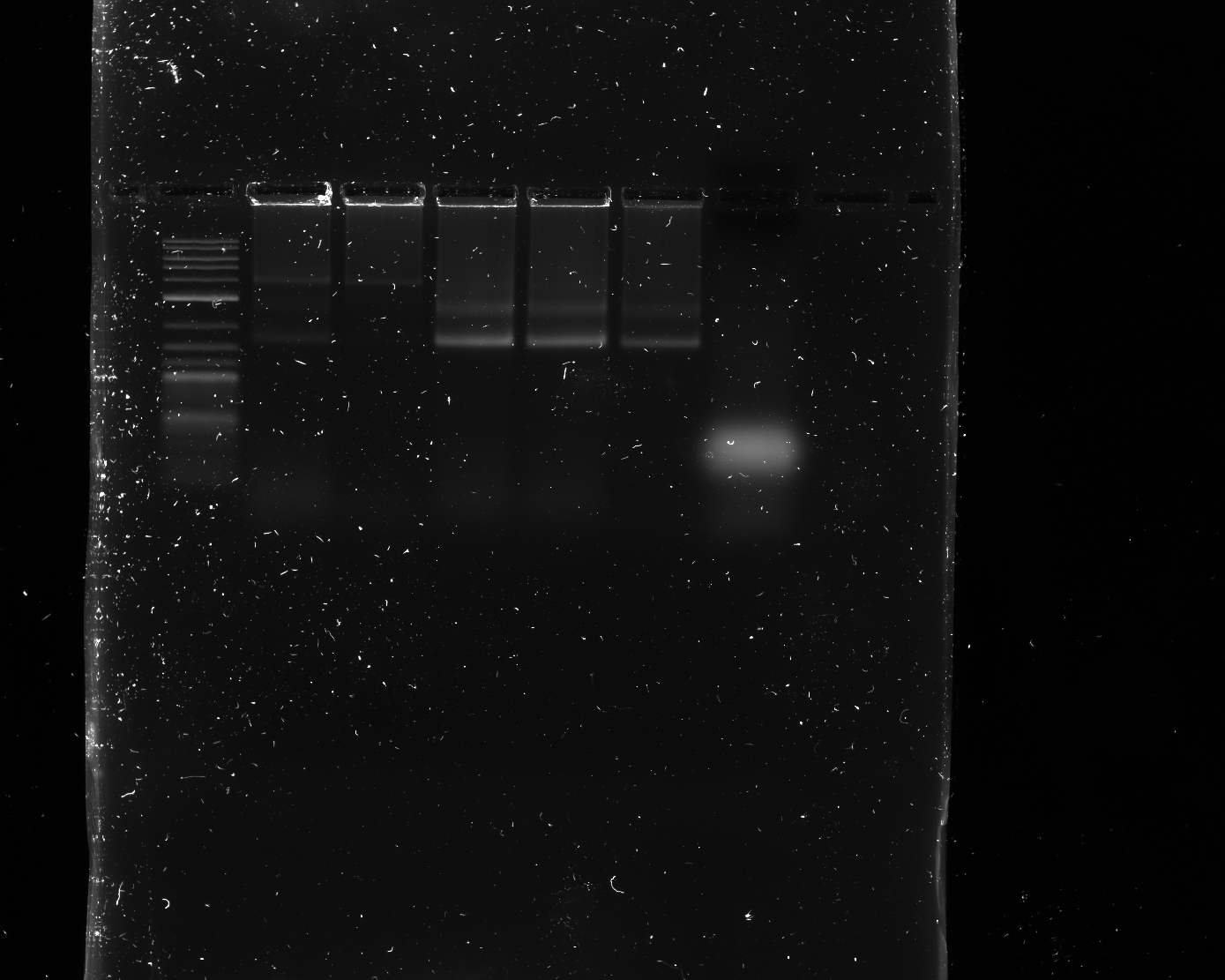

Supplement: Supplementary file 9 — Source Data [file 41467_2025_59189_MOESM9_ESM.zip › source_data/gels/fig_2a_3.tif]

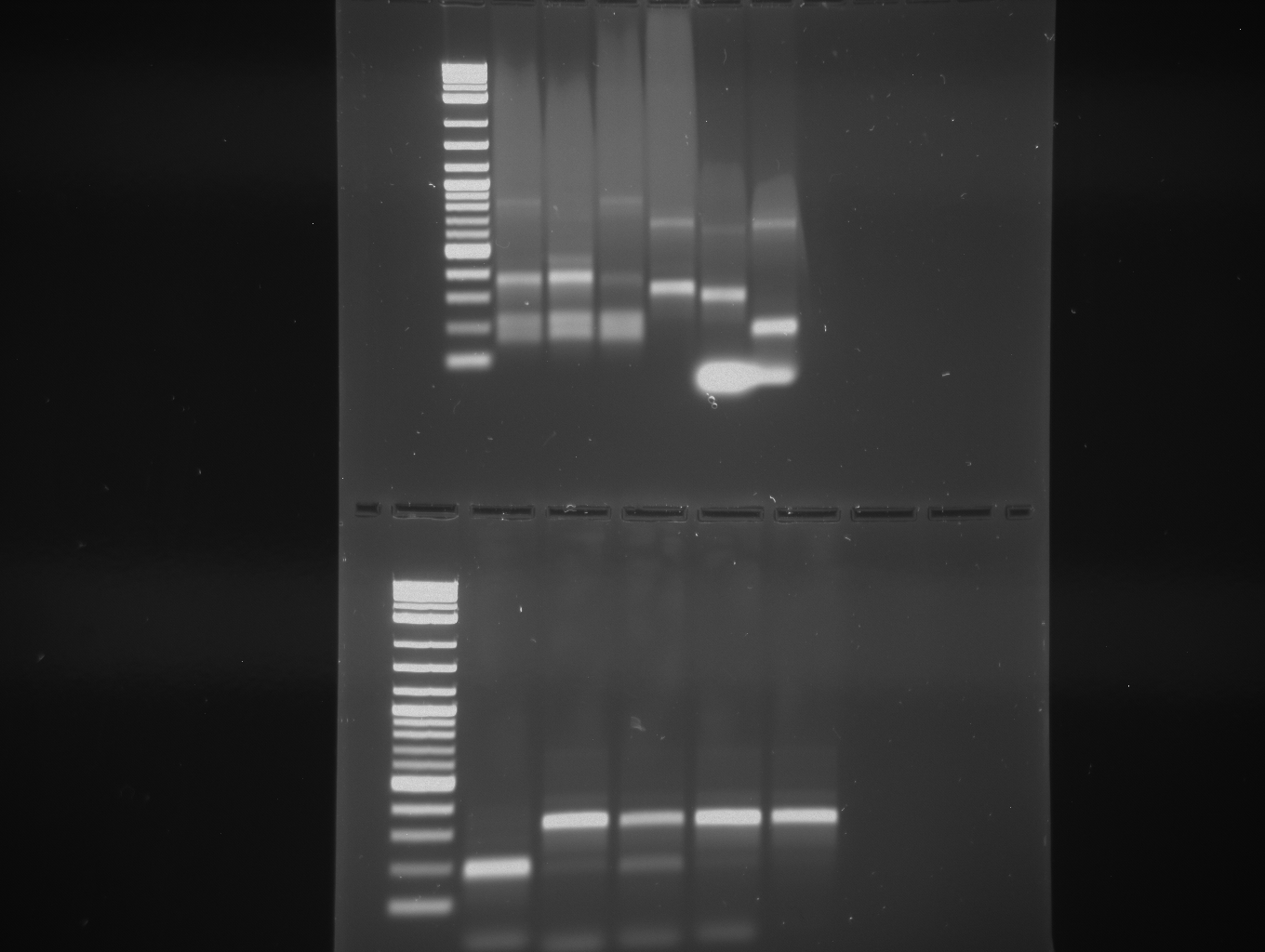

Supplement: Supplementary file 9 — Source Data [file 41467_2025_59189_MOESM9_ESM.zip › source_data/gels/fig_s14_2C.tif]

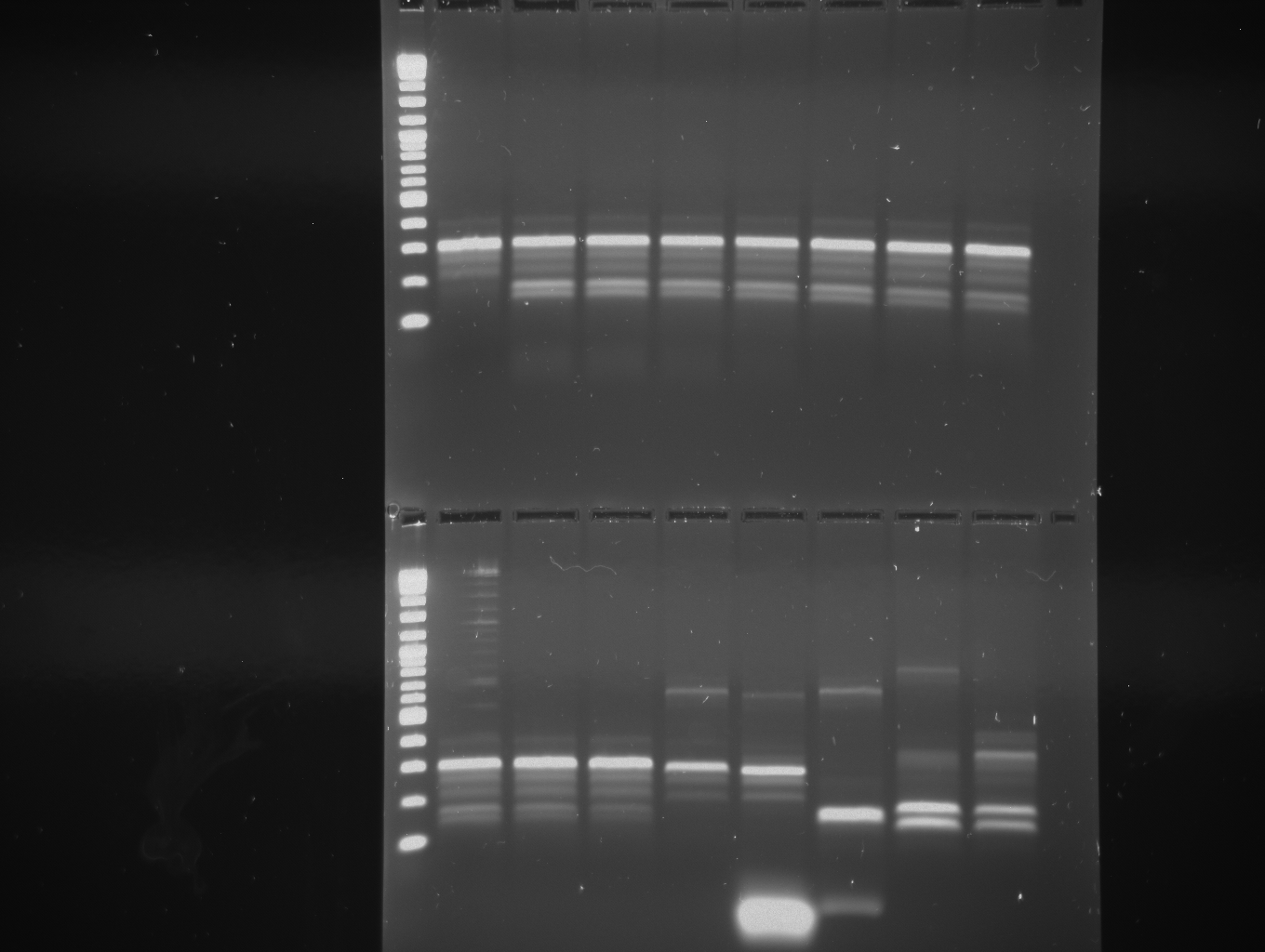

Supplement: Supplementary file 9 — Source Data [file 41467_2025_59189_MOESM9_ESM.zip › source_data/gels/fig_s14_2B.tif]

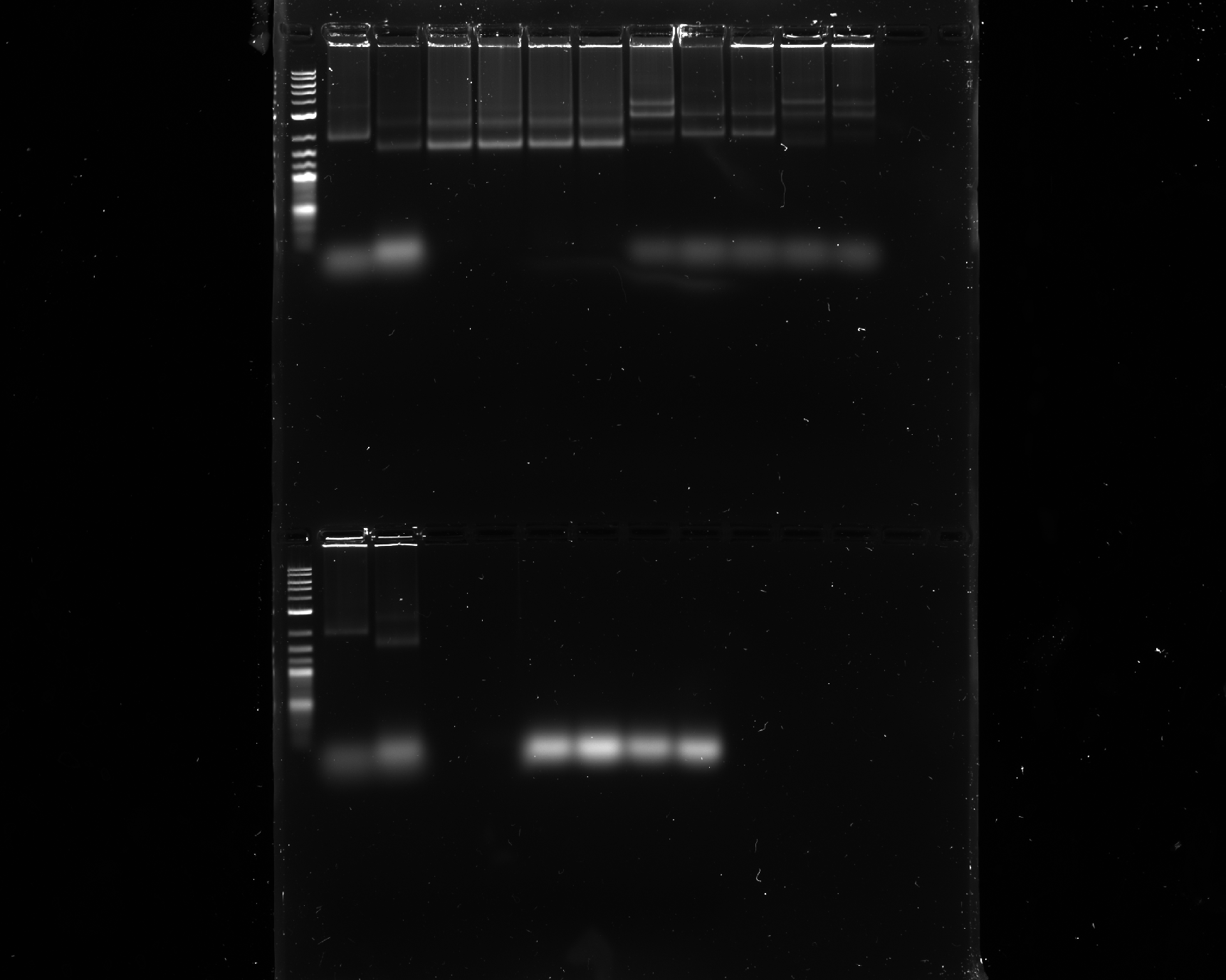

Supplement: Supplementary file 9 — Source Data [file 41467_2025_59189_MOESM9_ESM.zip › source_data/gels/fig_2b.tif]

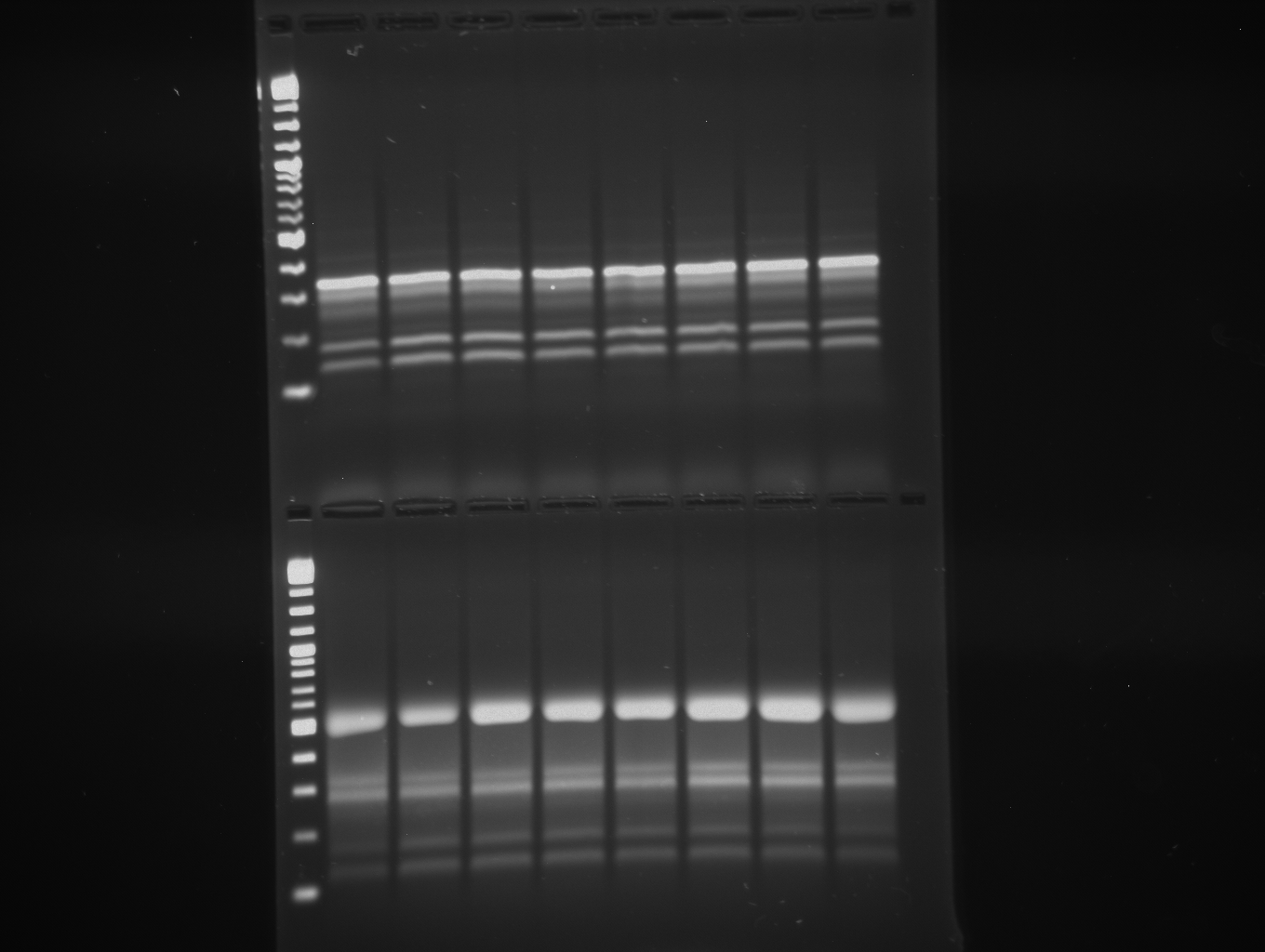

Supplement: Supplementary file 9 — Source Data [file 41467_2025_59189_MOESM9_ESM.zip › source_data/gels/fig_s14_S9A.tif]

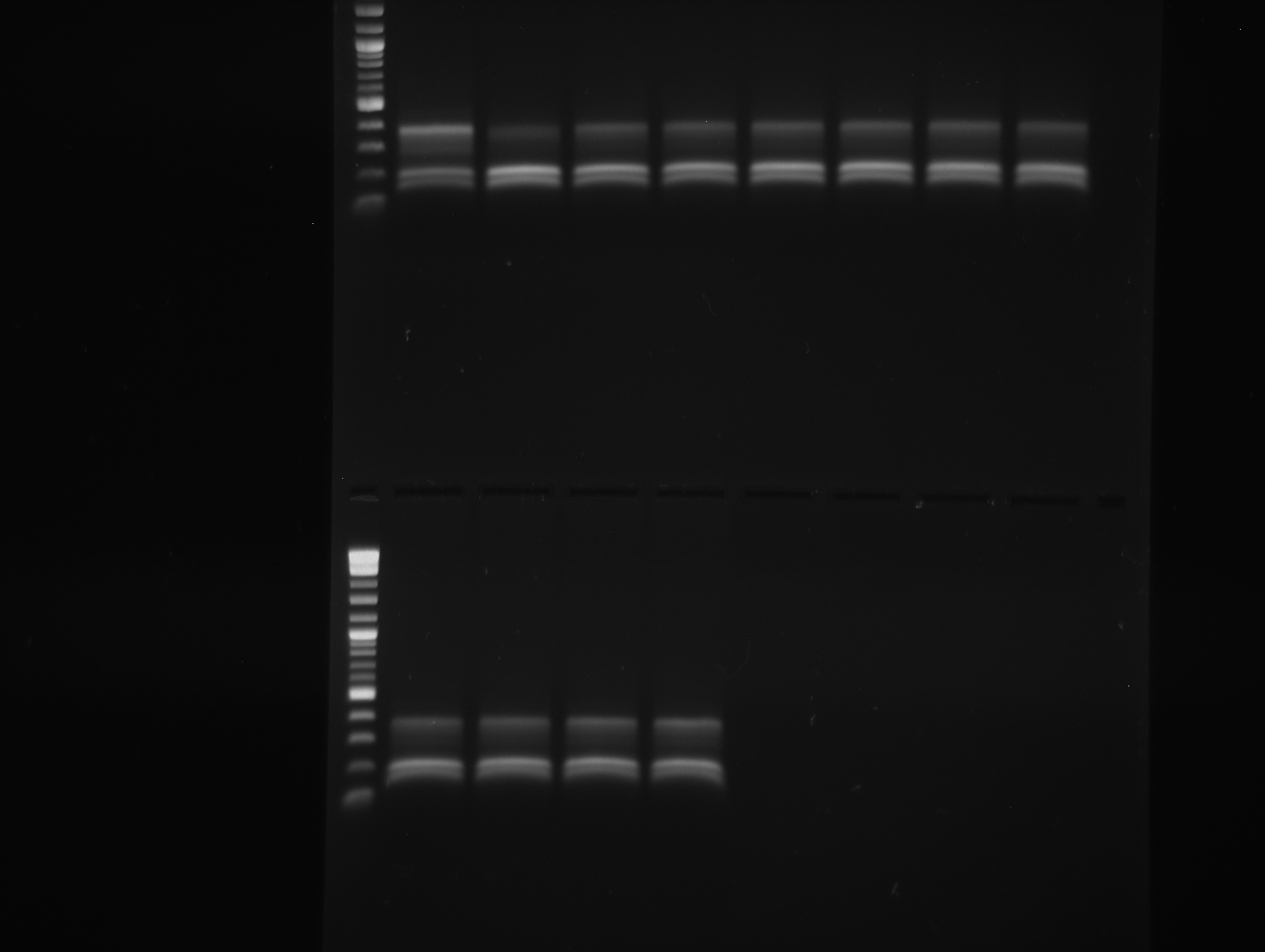

Supplement: Supplementary file 9 — Source Data [file 41467_2025_59189_MOESM9_ESM.zip › source_data/gels/fig_s14_2D.tif]

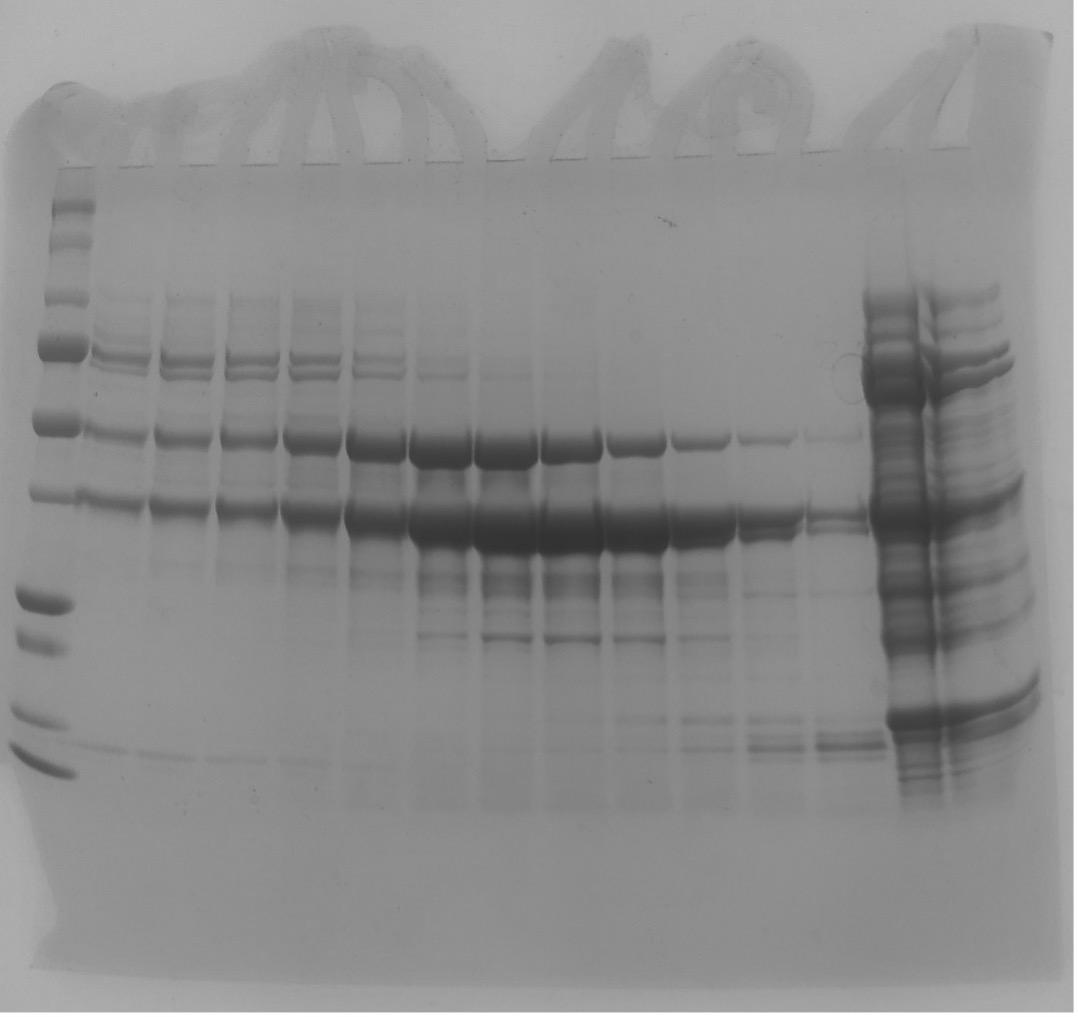

Supplement: Supplementary file 9 — Source Data [file 41467_2025_59189_MOESM9_ESM.zip › source_data/gels/fig_s9.tif]

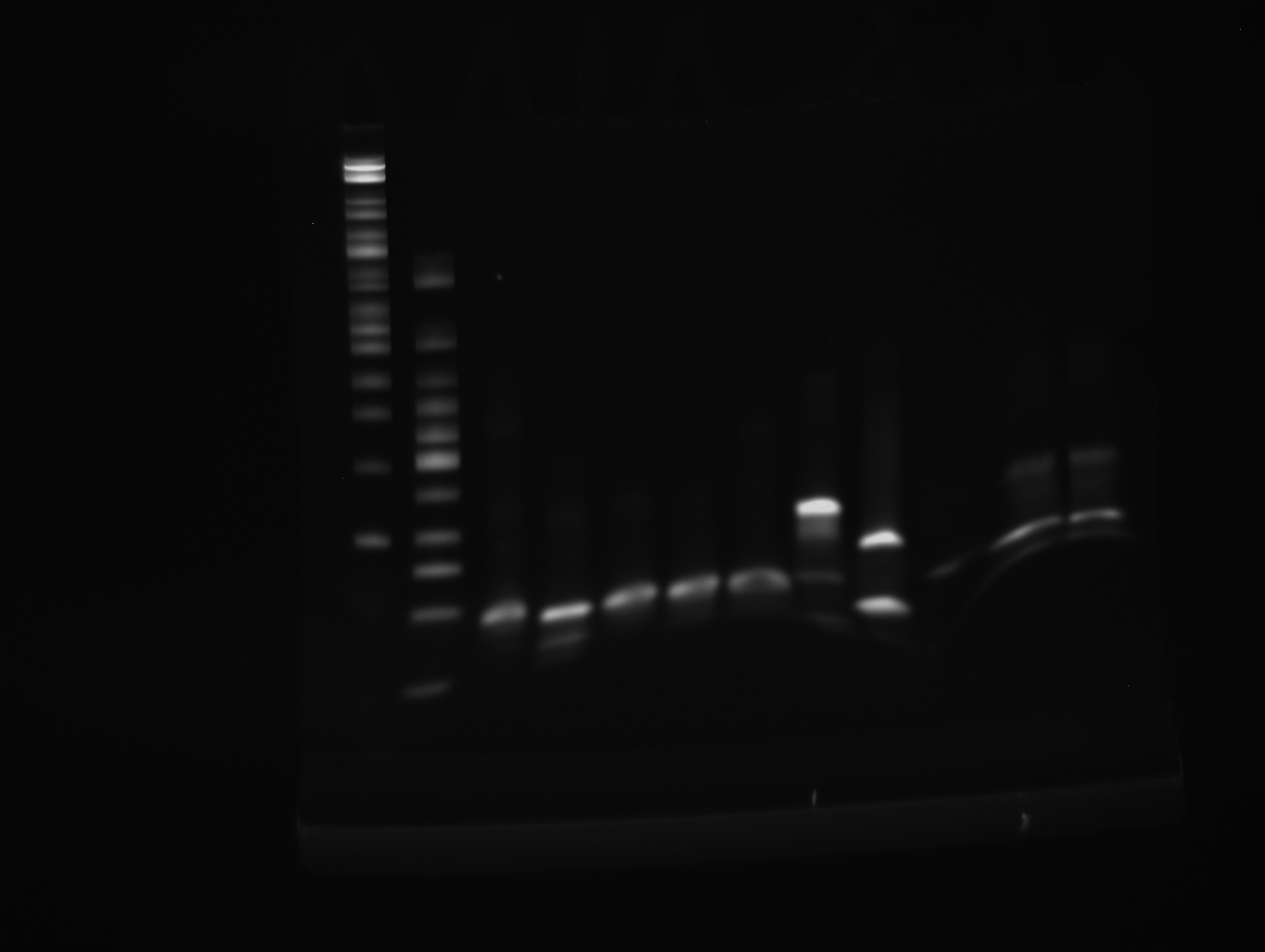

Supplement: Supplementary file 9 — Source Data [file 41467_2025_59189_MOESM9_ESM.zip › source_data/gels/fig_s7_2.tif]

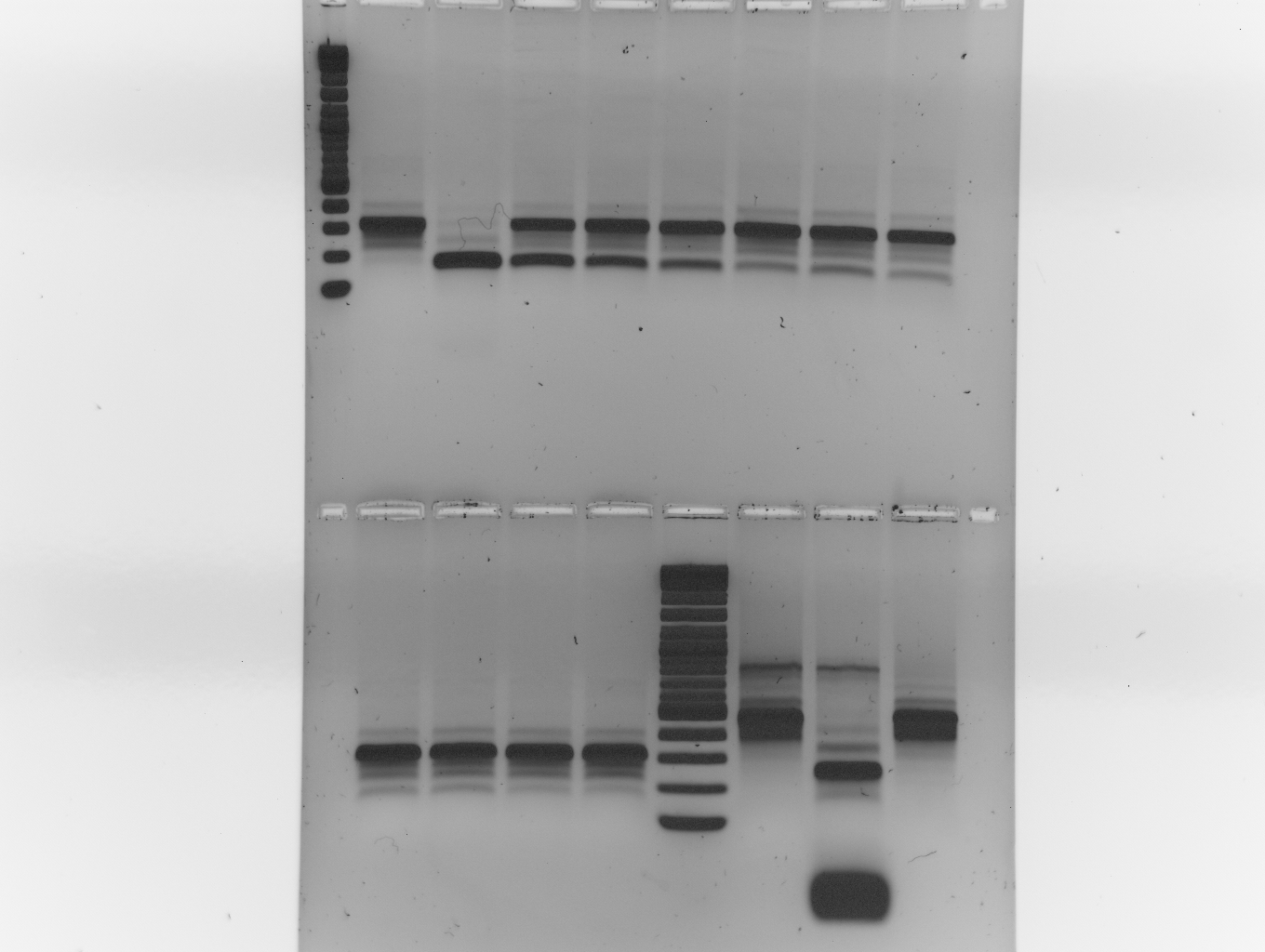

Supplement: Supplementary file 9 — Source Data [file 41467_2025_59189_MOESM9_ESM.zip › source_data/gels/fig_4a_1.tif]

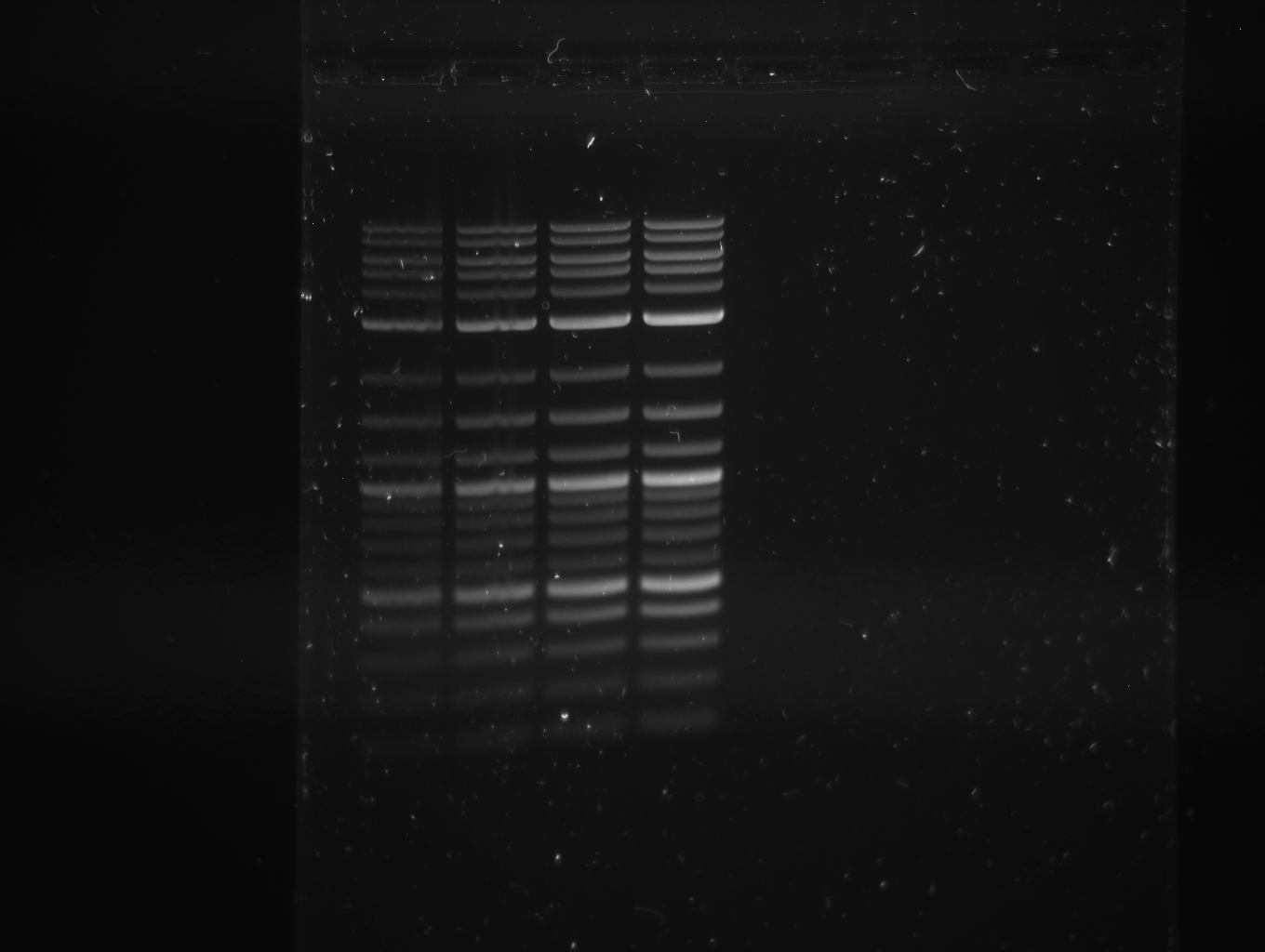

Supplement: Supplementary file 9 — Source Data [file 41467_2025_59189_MOESM9_ESM.zip › source_data/gels/fig_s5_1.tif]

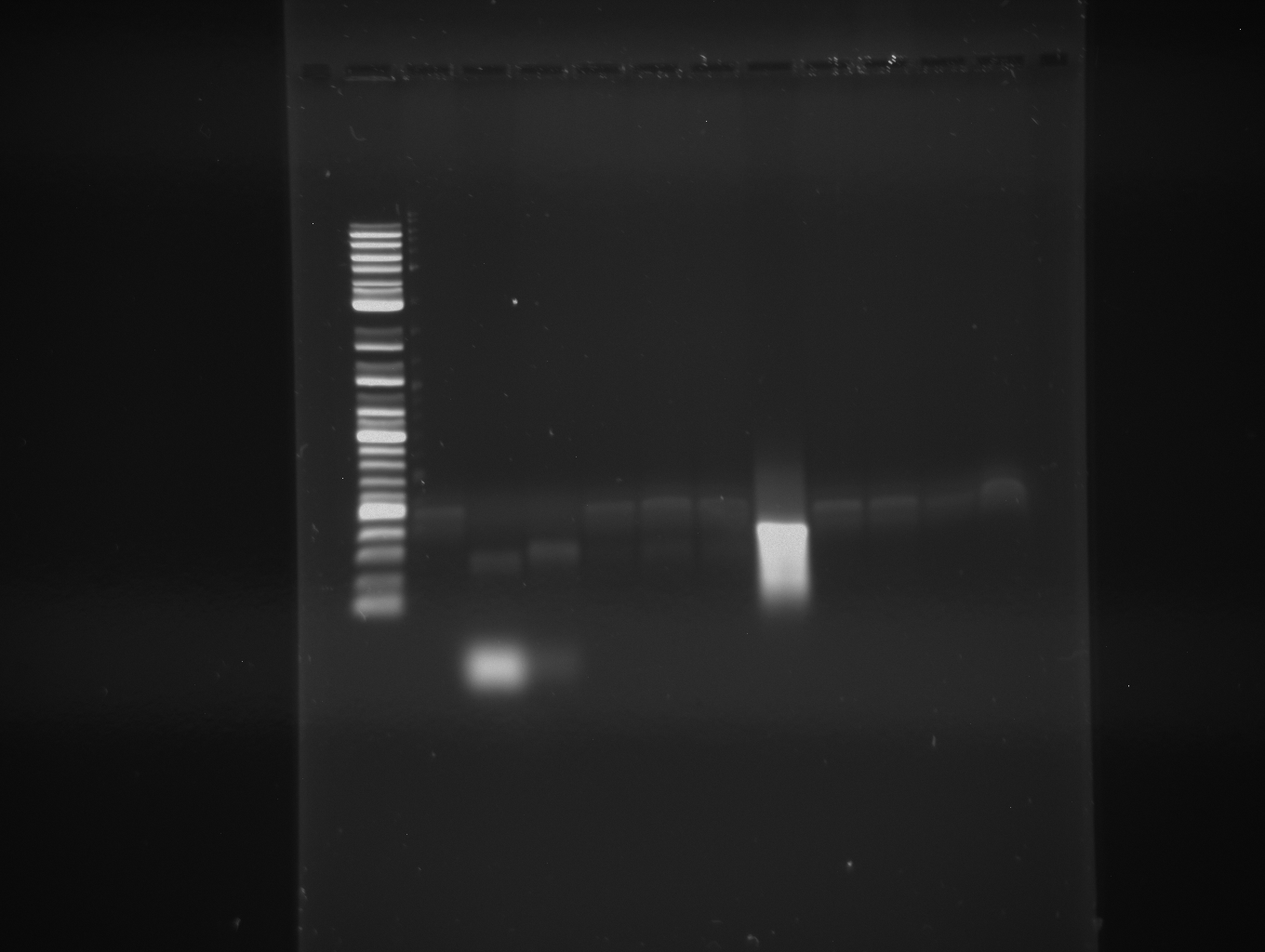

Supplement: Supplementary file 9 — Source Data [file 41467_2025_59189_MOESM9_ESM.zip › source_data/gels/fig_s7_1.tif]

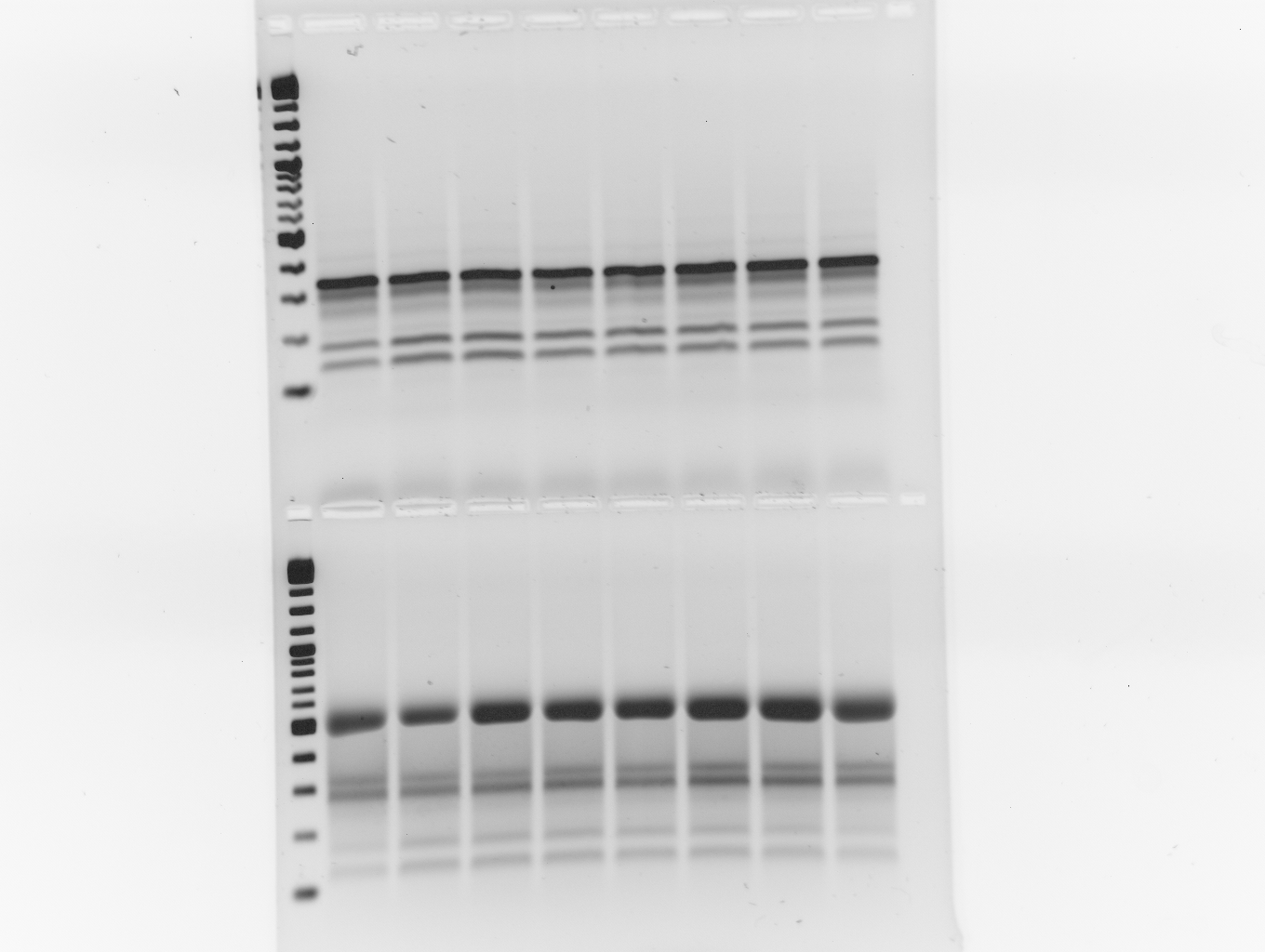

Supplement: Supplementary file 9 — Source Data [file 41467_2025_59189_MOESM9_ESM.zip › source_data/gels/fig_4a_2.tif]

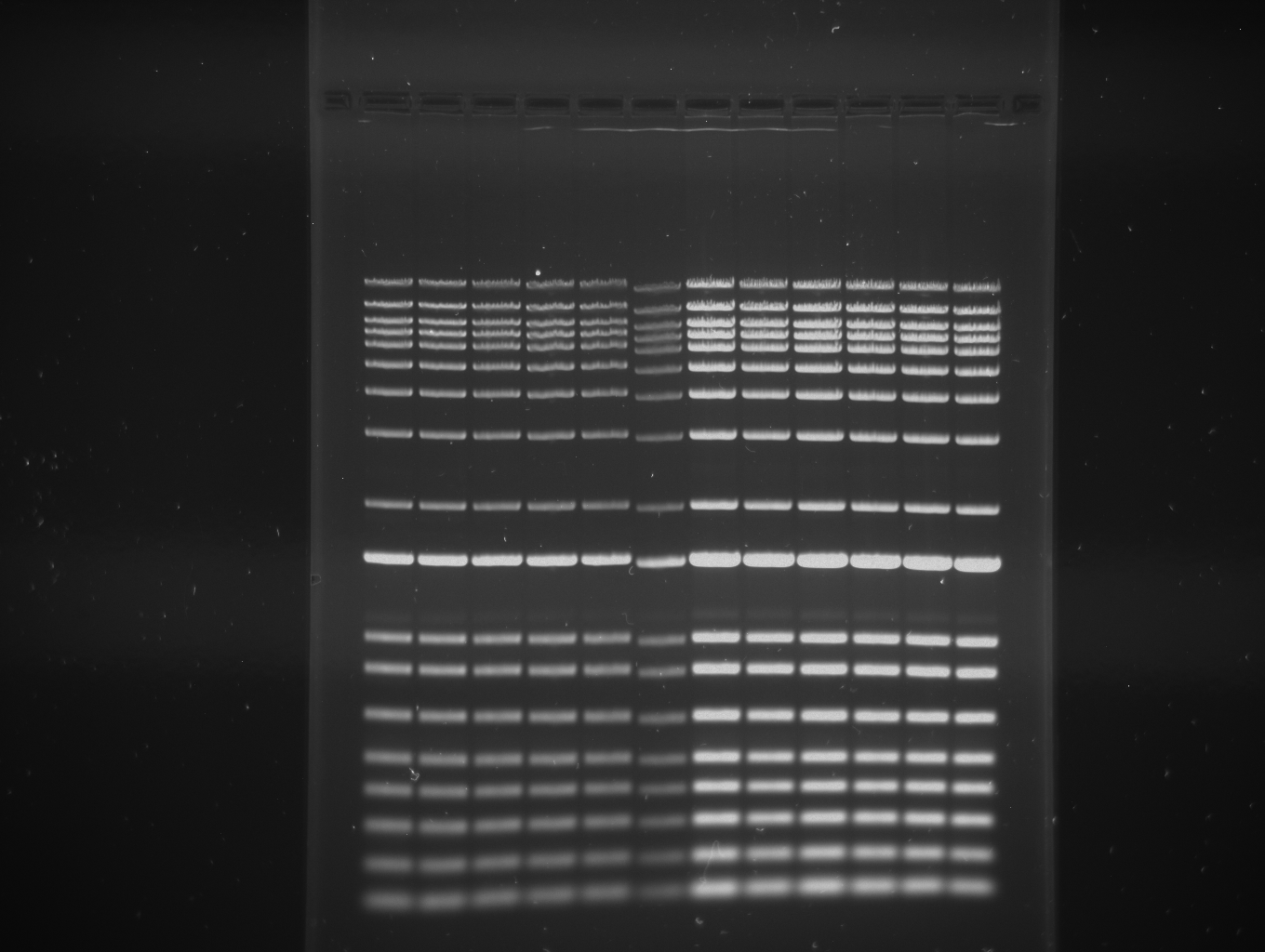

Supplement: Supplementary file 9 — Source Data [file 41467_2025_59189_MOESM9_ESM.zip › source_data/gels/fig_s5_2.tif]

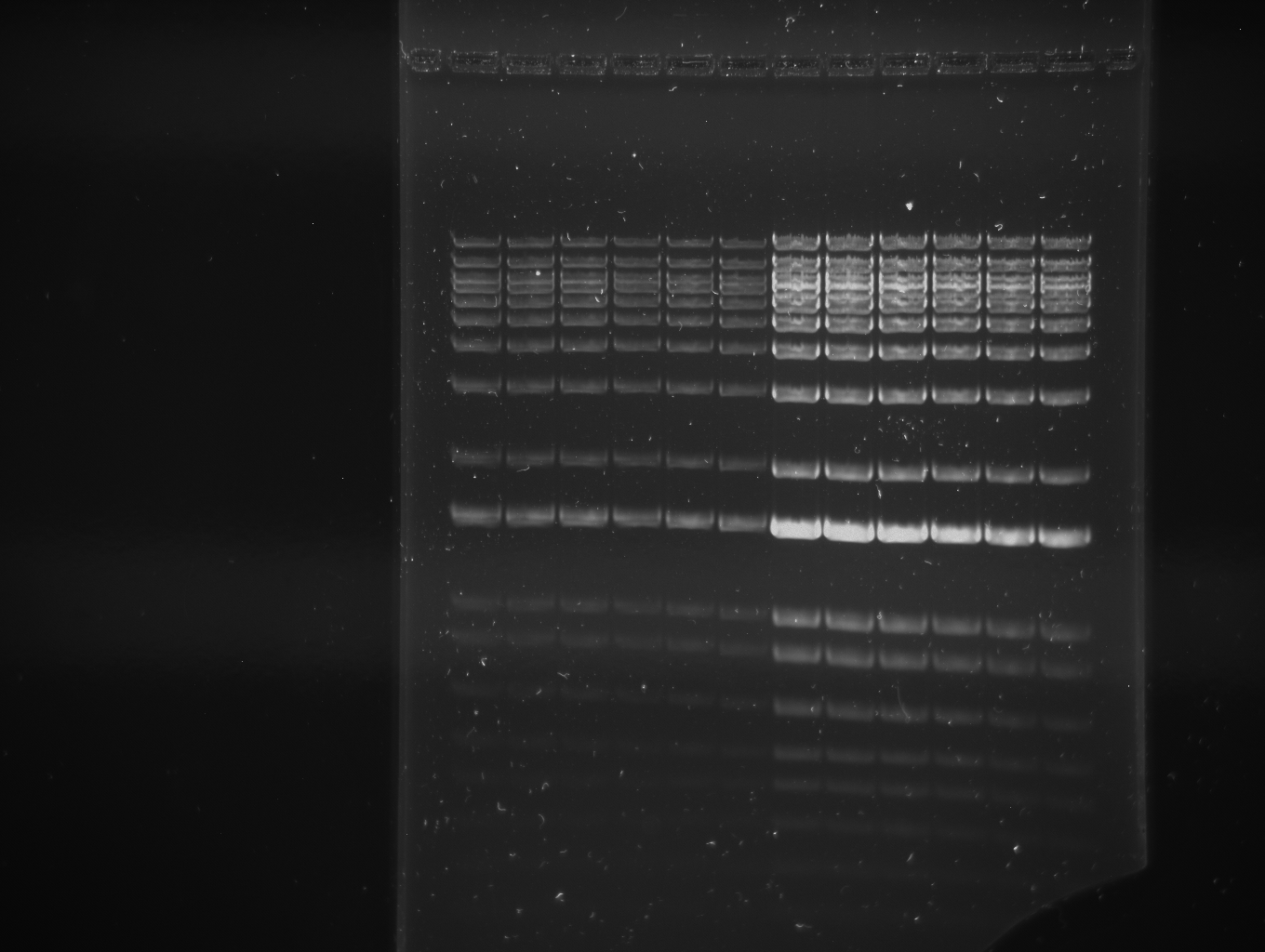

Supplement: Supplementary file 9 — Source Data [file 41467_2025_59189_MOESM9_ESM.zip › source_data/gels/fig_s1_2.tif]

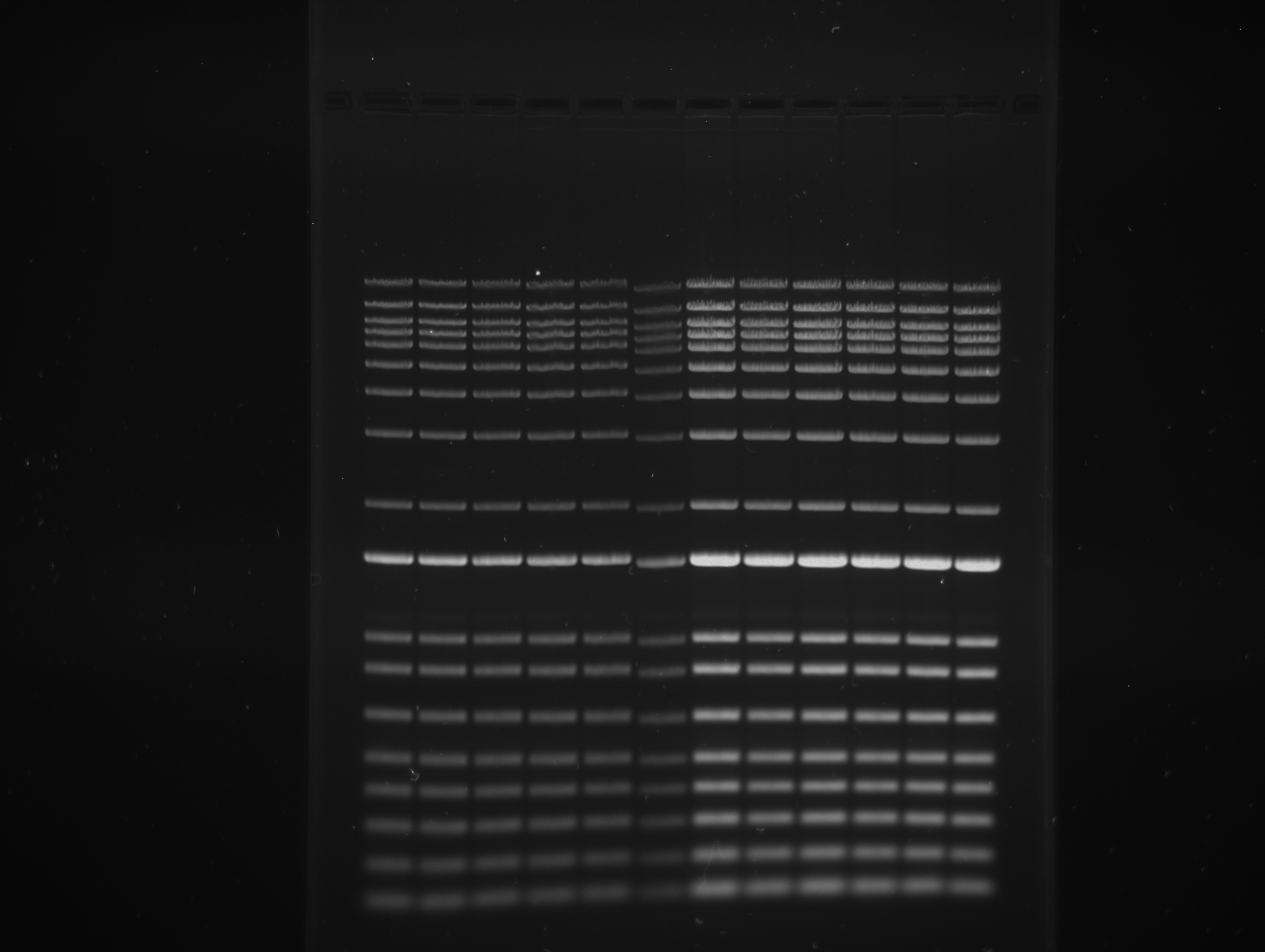

Supplement: Supplementary file 9 — Source Data [file 41467_2025_59189_MOESM9_ESM.zip › source_data/gels/fig_s1_1.tif]

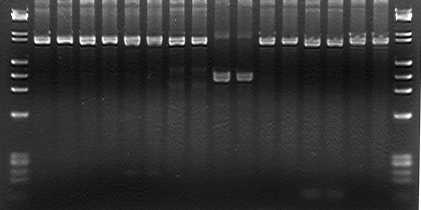

Supplement: Supplementary file 9 — Source Data [file 41467_2025_59189_MOESM9_ESM.zip › source_data/gels/fig_2a_1.JPG]
